# Association between hypertensive disorders of pregnancy and later risk of cardiovascular outcomes

**DOI:** 10.1186/s12916-021-02218-8

**Published:** 2022-01-25

**Authors:** Clare Oliver-Williams, David Stevens, Rupert A. Payne, Ian B. Wilkinson, Gordon C. S. Smith, Angela Wood

**Affiliations:** 1 British Heart Foundation Cardiovascular Epidemiology Unit, Department of Public Health and Primary Care, University of Cambridge, Cambridge, England UK; 2 Homerton College, Hills Road, Cambridge, UK; 3 Department of Health Sciences, University of Leicester, George Davies Centre, University Road, Leicester, UK; 4 Centre for Academic Primary Care, Population Health Sciences, University of Bristol, Bristol, UK; 5 Division of Experimental Medicine and Immunotherapeutics, Department of Medicine, University of Cambridge, Cambridge, England UK; 6 Cambridge Clinical Trials Unit, Cambridge University Hospitals NHS Foundation Trust, Addenbrooke’s Hospital, Cambridge, England UK; 7 Department of Obstetrics and Gynaecology, University of Cambridge, Cambridge, UK; 8 NIHR Cambridge Biomedical Research Centre, Cambridge, UK; 9 British Heart Foundation Centre of Research Excellence, University of Cambridge, Cambridge, England UK; 10 National Institute for Health Research Blood and Transplant Research Unit in Donor Health and Genomics, University of Cambridge, Cambridge, England UK; 11 National Institute for Health Research Cambridge Biomedical Research Centre, University of Cambridge and Cambridge University Hospitals, Cambridge, England UK; 12 Health Data Research UK Cambridge, Wellcome Genome Campus and University of Cambridge, Cambridge, England UK

**Keywords:** Gestational hypertension, Pre-eclampsia, Cardiovascular disease, Women, Pregnancy

## Abstract

**Background:**

Hypertensive disorders of pregnancy are common pregnancy complications that are associated with greater cardiovascular disease risk for mothers. However, risk of cardiovascular disease subtypes associated with gestational hypertension or pre-eclampsia is unclear. The present study aims to compare the risk of cardiovascular disease outcomes for women with and without a history of gestational hypertension and pre-eclampsia using national hospital admissions data.

**Methods:**

This was a retrospective cohort study of national medical records from all National Health Service hospitals in England. Women who had one or more singleton live births in England between 1997 and 2015 were included in the analysis. Risk of total cardiovascular disease and 19 pre-specified cardiovascular disease subtypes, including stroke, coronary heart disease, cardiomyopathy and peripheral arterial disease, was calculated separately for women with a history of gestational hypertension and pre-eclampsia compared to normotensive pregnancies.

**Results:**

Amongst 2,359,386 first live births, there were 85,277 and 74,542 hospital admissions with a diagnosis of gestational hypertension and pre-eclampsia, respectively. During 18 years (16,309,386 person-years) of follow-up, the number and incidence of total CVD for normotensive women, women with prior gestational hypertension and women with prior pre-eclampsia were *n* = 8668, 57.1 (95% CI: 55.9–58.3) per 100,000 person-years; *n* = 521, 85.8 (78.6–93.5) per 100,000 person-years; and *n* = 518, 99.3 (90.9–108.2) per 100,000 person-years, respectively. Adjusted HRs (aHR) for total CVD were aHR (95% CI) = 1.45 (1.33–1.59) for women with prior gestational hypertension and aHR = 1.62 (1.48–1.78) for women with prior pre-eclampsia.

Gestational hypertension was strongly associated with dilated cardiomyopathy, aHR = 2.85 (1.67–4.86), and unstable angina, aHR = 1.92 (1.33–2.77). Pre-eclampsia was strongly associated with hypertrophic cardiomyopathy, aHR = 3.27 (1.49–7.19), and acute myocardial infarction, aHR = 2.46 (1.72–3.53). Associations were broadly homogenous across cardiovascular disease subtypes and increased with a greater number of affected pregnancies.

**Conclusions:**

Women with either previous gestational hypertension or pre-eclampsia are at greater risk of a range of cardiovascular outcomes. These women may benefit from clinical risk assessment or early interventions to mitigate their greater risk of various cardiovascular outcomes.

**Supplementary Information:**

The online version contains supplementary material available at 10.1186/s12916-021-02218-8.

## Background

Pregnancy poses substantial challenges to the maternal cardiovascular system, which can reveal themselves through pregnancy complications that can have longer term effects on maternal health for decades after pregnancy [1]. Hypertensive disorders of pregnancy, including pre-eclampsia (PE) and gestational hypertension (GH), are characterised by new hypertension after 20 weeks’ gestation and, in the case of PE, with additional abnormal findings related to end-organ damage [2]. Gestational hypertension complicates approximately 15% of all pregnancies, of which up to 30% are further complicated by PE [2]. Both GH and PE can cause significant morbidity and mortality for both mother and foetus [3]. UK and US studies reported that hypertensive conditions in pregnancy caused a third of severe maternal morbidity [4, 5] and over 7% of mothers in the UK and Norway who had a stillbirth had pregnancy-induced hypertension [3, 6].

Cardiovascular disease (CVD) is a leading cause of morbidity and mortality for women globally. Women may have different symptoms of CVD compared to men [7], which can impair the speed at which treatment is provided [8]. This emphasises the need to identify women at risk of CVD early in life to ensure prevention is appropriately and effectively targeted. PE is associated with CVD in the decades after pregnancy, with an approximate 2-fold higher risk of coronary heart disease (CHD) (relative risks [RR] of 2.33–2.47), cerebrovascular disease (RR of 1.76–2.03), peripheral arterial disease (RR of 1.87) and CVD mortality (RR of 2.29) relative to women with normotensive pregnancies [9, 10]. The mechanism underlying this is not fully understood, but it is thought that shared risk factors may link PE and CVD [11]. It is less clear if GH may also be associated with greater CVD risk, with evidence limited to a few outcomes including CHD [12, 13], stroke [12–14], chronic hypertension [13] and cardiomyopathy [15]. This lack of clarity on the long-term outcomes for women with GH has led to calls for further and more detailed research [16].

This study aimed to address this uncertainty by characterising the risk of a range of pre-specified cardiovascular subtypes separately for women with a history of GH or PE using a nationwide hospital records cohort study. It was hypothesised that women with a history of GH or PE are at greater risk of multiple cardiovascular outcomes in the decades after pregnancy and that the magnitude of the associations increases with the number of affected pregnancies.

## Results

The cohort consisted of 2,359,386 women with 4,033,658 live births during 1997-2015 (Additional file 1: Fig. S3). There were 115,183 first deliveries with a GH diagnosis and 92,688 first deliveries with a PE diagnosis. The mean maternal age at first delivery was 26.9 years (SD = 5.8). 0.2% of 2,359,386 women at first delivery had a reported diabetes diagnosis, 72.9% had reported white ethnicity and 13.2% were classified as being in the most deprived 10% of the deprivation index (Additional file 1: Table S2). The mean maternal age at all deliveries, including first and subsequent deliveries, was 28.1 years (SD = 5.7) (Additional file 1: Table S3). Women with 1 or more pregnancies complicated by GH or PE were more likely to have a reported diabetes diagnosis (Additional file 1: Tables S2 and S3). Women were followed up to a maximum of 18 years after their first live-birth delivery. During 16.3 million person-years (median 2.3 years of follow-up [5^th^–95^th^ percentiles 0.3–12.1]), there were 10,806 first incident CVD outcomes, including 2974 coronary heart disease events, 2131 strokes, 58 abdominal aortic aneurysms and 389 peripheral arterial disease events (Additional file 1: Table S2).

### Incidence of hypertensive disorders of pregnancy (HDP) and CVD

**Fig. 1:**
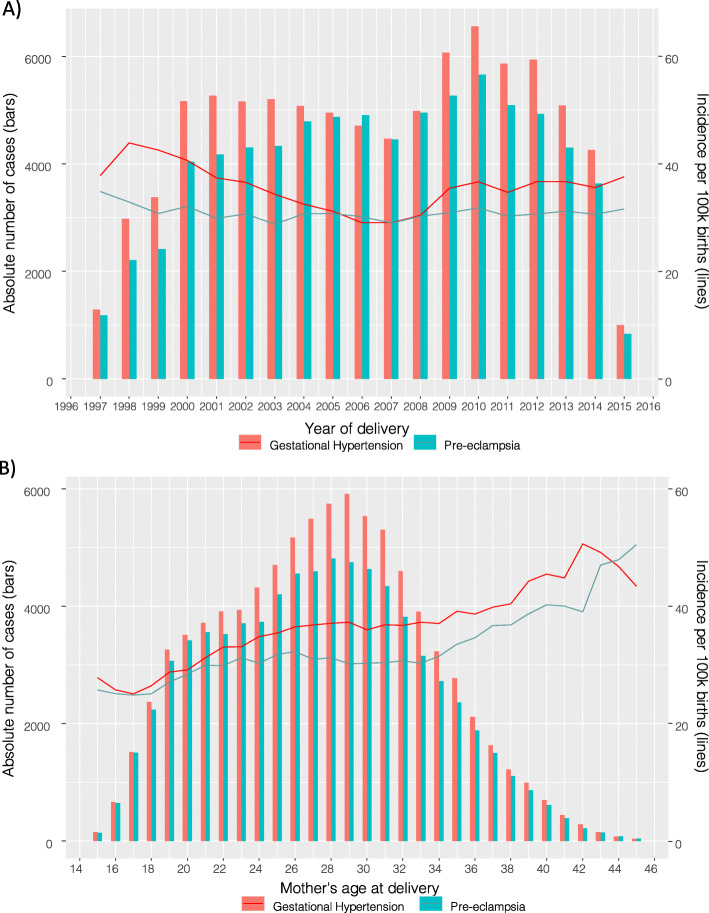
Incidence over **A** time and **B** age of gestational hypertension and pre-eclampsia amongst nulliparous women. *Bars:* incidence amongst nulliparous women; *solid lines*: incidence per 100,000 person-years; *dotted lines*: 95% confidence intervals. The years 1997 and 2015 were excluded from the plot as data only covered part of these years. There was less available data for 1998 and 1999 due to the parity of the pregnancies being recorded less consistently in that period

The crude incidence of GH decreased over the study period, before increasing again to 3.7% by 2014, while the crude incidence of PE remained stable (Fig. 1A). By maternal age at delivery, the crude incidence of both GH and PE in nulliparous women increased with age to 5.3% and 4.0% respectively for mothers aged 42, although the absolute number of GH and PE cases amongst nulliparous women was highest amongst women aged between 27 and 30 years (Fig. 1B).

**Fig. 2:**
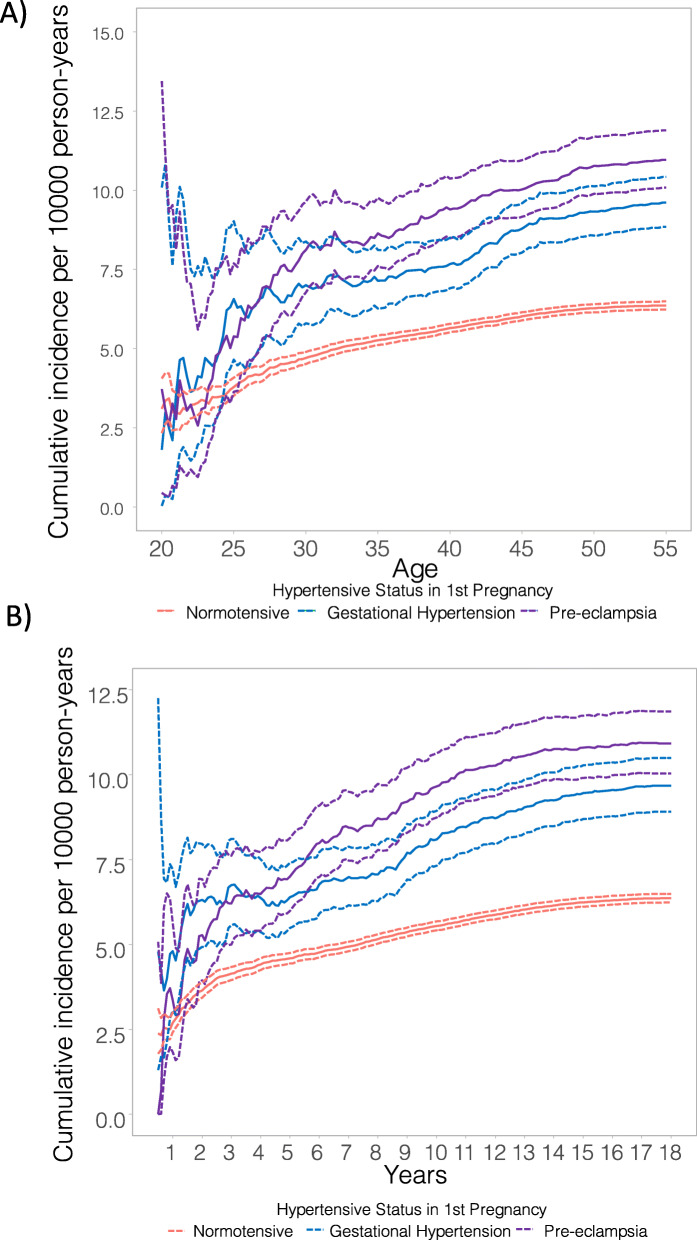
Cumulative incidence of all-cause cardiovascular disease by **A** year and **B** age after first delivery by hypertensive status

Compared to women with a normotensive first pregnancy, the cumulative incidence of total CVD is higher amongst women with GH and PE by time since first pregnancy (Fig. 2A) and by age (Fig. 2B). The absolute risk of total CVD in the 5 years after first delivery is 39.2, 54.7 and 62.6 per 100,000 person-years for women with normotensive first pregnancy, GH and PE in the first pregnancy, respectively. The most common first incident CVD events were atrial fibrillation and flutter for women with a normotensive first pregnancy and for women with GH in their first pregnancy (Additional file 1: Fig. S4). The most common first incident CVD event for women with PE in their first pregnancy was CHD.

### Risk of total CVD by history of HDP

**Fig. 3:**
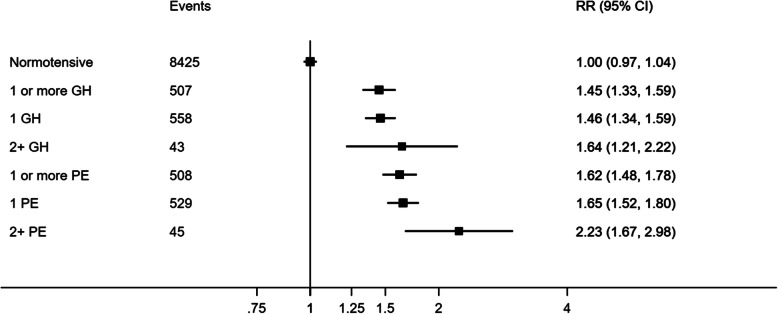
Risk of all-cause cardiovascular disease by the number of pregnancies complicated by gestational hypertension or pre-eclampsia, compared to women with normotensive pregnancies. GH, gestational hypertension; PE, pre-eclampsia. Adjusted for maternal age at delivery, socioeconomic status, ethnicity and diabetes

In age-adjusted Cox models, risk of total CVD was greater for women with history of GH or PE compared with women with a history of only normotensive pregnancies: HR = 1.44 (95%CI: 1.32, 1.57) for GH and HR = 1.67 (95%CI: 1.52, 1.82) for PE (Fig. 3). These HRs were unaltered by adjustment for additional covariates, HR = 1.45 (95%CI: 1.33, 1.59) for GH and HR = 1.62 (95%CI: 1.48, 1.78) for PE (Additional file 1: Tables S4 and S5). The risk of total CVD was higher amongst women with at least two pregnancies affected by GH or PE, HR = 1.64 (95%CI: 1.21, 2.21) and HR = 2.23 (95%CI: 1.76, 2.99), respectively (Fig. 3).

The risk of CVD associated with GH was broadly consistent over 18 years of follow-up (Additional file 1: Fig. S5). In contrast, the CVD risk associated with PE increased over time (Additional file 1: Fig. S6).

### Risk of pre-specified CVD subtypes by history of HDP

**Fig. 4:**
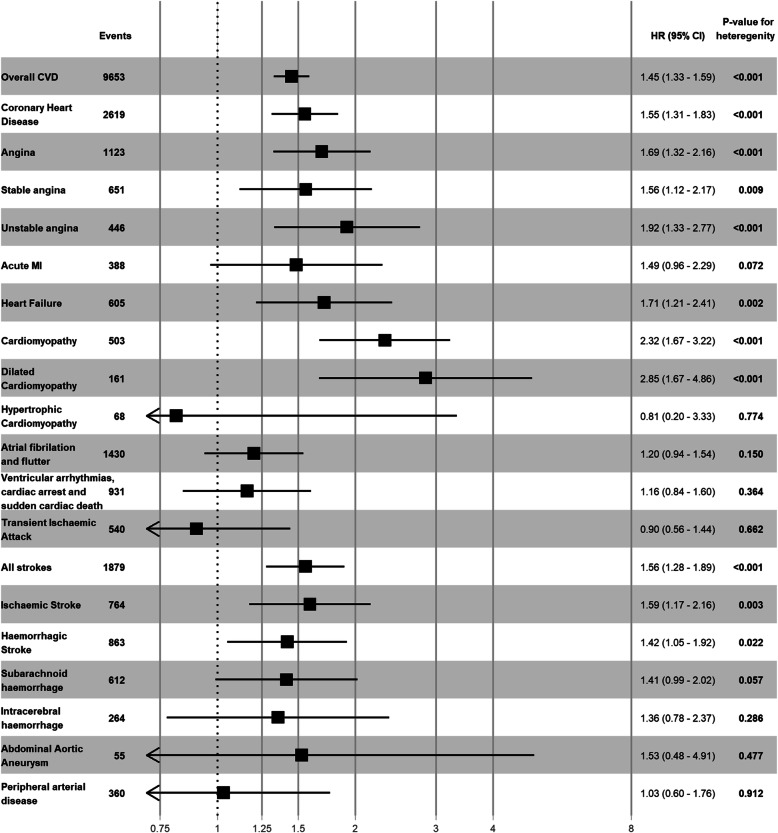
Risk of cardiovascular subtypes for women with gestational hypertension in their first pregnancy compared to women with normotensive pregnancies. CVD, cardiovascular disease; GH, gestational hypertension; MI - myocardial infarction. Adjusted for maternal age at delivery, socioeconomic status, ethnicity and diabetes

Women with GH in their first pregnancy were at a higher risk of coronary heart disease (HR = 1.55 (95%CI: 1.31, 1.83)), stable angina (HR = 1.56 (95%CI: 1.12, 2.17)), unstable angina (HR = 1.92 (95%CI: 1.33, 2.77)), heart failure (HR = 1.71 (95%CI: 1.21, 2.41)), dilated cardiomyopathy (HR = 2.85 (95%CI: 1.67, 4.86)), ischemic stroke (HR = 1.59 (95%CI: 1.17, 2.16)) and hemorrhagic stroke (HR = 1.42 (95%CI: 1.05, 1.92)) (Fig. 4, Additional file 1: Table S4). Women with GH in their first pregnancy were at more than a 2-fold higher risk of dilated cardiomyopathy. The risk of CHD and all strokes increased with the number of pregnancies affected by GH (Additional file 1: Fig. S7, Table S6). Women with two GH pregnancies were at approximately twice the risk of CHD and stroke compared to women with normotensive pregnancies, HR = 1.93 (95%CI: 1.16–3.20) and HR = 2.16 (95%CI: 1.19–3.90), respectively.

**Fig. 5:**
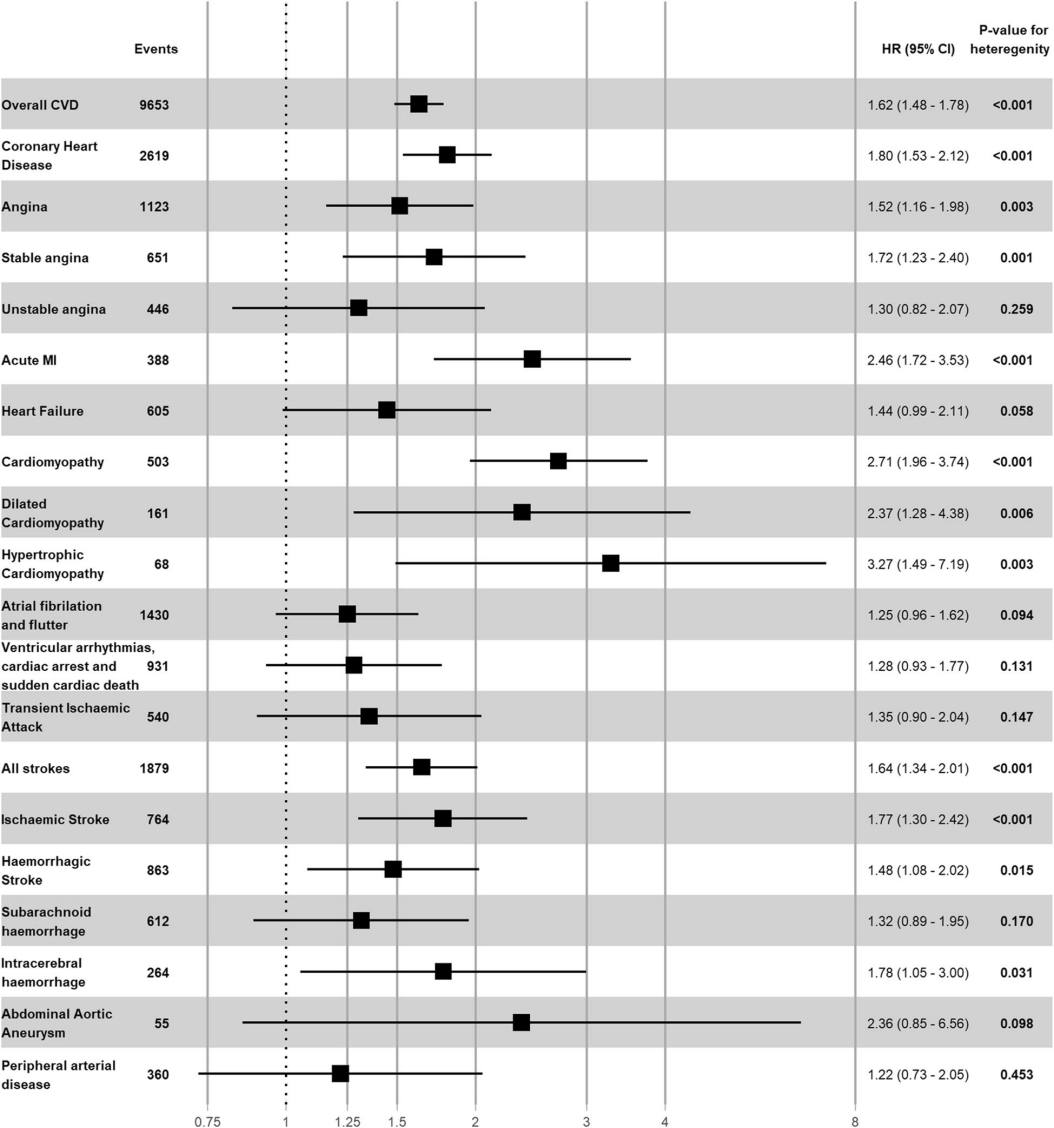
Risk of pre-specified cardiovascular subtypes for women with pre-eclampsia in their first pregnancy compared to women with normotensive pregnancies. CVD - cardiovascular disease; MI - myocardial infarction; PE, pre-eclampsia. Adjusted for maternal age at delivery, socioeconomic status, ethnicity and diabetes

Women with PE in their first pregnancy were at a higher risk of coronary heart disease (HR = 1.62 (95%CI: 1.48, 1.78)), stable angina (HR = 1.72 (95%CI: 1.23, 2.40)), acute myocardial infarction (HR = 2.46 (95%CI: 1.72, 3.53)), dilated cardiomyopathy (HR = 2.37 (95%CI: 1.28, 4.38)), hypertrophic cardiomyopathy (HR = 3.27 (95%CI: 1.49, 7.19)), ischemic stroke (HR = 1.77 (95%CI: 1.30, 2.42)) and intracerebral haemorrhage (HR = 1.78 (95%CI: 1.05, 3.00)), and associations were generally stronger than those observed with GH (Fig. 5, Additional file 1: Table S5). Women with PE in their first pregnancy were at more than a 2-fold higher risk of acute myocardial infarction, dilated cardiomyopathy and hypertrophic cardiomyopathy. The risk of CHD and all strokes increased with the number of pregnancies affected by PE (Additional file 1: Fig. S8, Table S7). Women with two PE pregnancies were at more than twice the risk of CHD and stroke compared to women with normotensive pregnancies, HR = 2.81 (95%CI: 1.75, 4.53) and HR = 2.35 (95%CI: 1.22, 4.53), respectively.

### Risk of pre-specified CVD subtypes by pre-eclampsia severity

The association with total CVD was greater with more severe PE in the first pregnancy; risk was highest for women with severe PE, HR = 1.91 (95%CI: 1.57, 2.32) (Additional file 1: Fig. S9, Table S8). Risk increased with the severity of PE for CVD, specifically, CHD, cardiomyopathy, and all strokes, specifically ischemic stroke and intracerebral haemorrhage.

### Sensitivity analysis

When the start of follow-up was changed to either 3 months post-partum or 12 months post-partum, the findings were consistent with the main findings for nulliparous women (Additional file 1: Tables S9 and S10).

## Discussion

This retrospective cohort study of nearly 2.5 million women with first pregnancies provides strong evidence that women with a history of either GH or PE are at higher risk of a range different CVD subtypes. No previous cohort study has combined long follow-up and a large enough sample to fully investigate the association between HDP and a range of CVD subtypes. To our knowledge, novel associations of GH with stable and unstable angina and PE with stable angina and transient ischemic attack have not been previously shown. Our analyses also suggested that women with a history of PE were at slightly higher risk of CVD outcomes than women with GH. Further, there was evidence of a dose-response relationship; women with more than one pregnancy affected by GH or PE and women with severe PE had the highest risks of several CVD subtypes.

Our study has highlighted that positive associations between GH, PE and total CVD risk exist across a range of several, distinct, pre-specified CVD subtypes. Two theories of the potential mechanisms underlying the relationship between HDP and CVD exist. First, shared genetic or pre-conception lifestyle risk factors (e.g. leading to raised blood pressure and body mass index [BMI]) may increase the risks of both HDP and CVD subtypes [17]. For example, our finding of a strong association between PE (but not GH) and the genetic condition hypertrophic cardiomyopathy supports this theory [18]. Second, greater CVD risk may be a result of vascular damage (e.g. blood vessel function [19] and cardiac structure and function [20, 21]) in HDP complicated pregnancies, arising from inflammatory stress, coagulation dysregulation and endothelial damage [22]. Our dose-effect findings in this study, whereby risk is greater with recurrent HDP, supports this theory. It is likely both mechanistic theories contribute to the greater risks across the range of CVD subtypes. Further, if pregnancy is viewed as a “stress test”, as is often espoused, then onset of a HDP of pregnancy in the second rather than third trimester should be associated with higher CVD risk, as it indicates a “failed” stress test at lower physiological demands. Previous work supports this, with greater changes in angiogenic factors in women with preterm PE compared to term PE [23]. Similarly, our findings indicate higher CVD risks amongst women with severe PE compared to moderate and mild PE.

Our study’s findings have implications for public health recommendations to monitor CVD risk amongst women following pregnancy with GH. Currently, the American Heart Association recommend monitoring the cardiovascular risk of women with previous PE but not GH [24], despite low absolute risks. Although it is unclear how many women do receive follow-up. In our study, the absolute risk of total CVD for women with PE in the 5 and 10 years after diagnosis is 62.6 and 86.8 per 100,000 person-years respectively; in comparison, the absolute risk for women in the 5 and 10 years after GH diagnosis is 54.7 and 72.7 per 100,000 person-years, respectively.

The associations across a range of acute and chronic cardiovascular diseases in patients with GH or PE also have implications for the delivery of preventive strategies to lower CVD risk in women. Diagnosis of HDP highlights a woman’s risk of CVD early in life. These women may benefit from counselling during and/or after an affected pregnancy, and lifestyle changes, such as those that limit interpregnancy weight gain[25].

Our study used nation-wide electronic health records, which has a number of advantages. It allowed for differentiation of a wide range of cardiovascular diseases, assessment of prevalence of HDP over time and age. It provided long-term follow-up (up to 18 years), allowing assessment of the national incidence of GH and PE over time. We were able to assess diagnosed pregnancy complications, rather than self-reported, and assess risk after both affected first pregnancies, and multiple affected pregnancies. The generalisability of the findings was enhanced by inclusion of population-wide data.

Nevertheless, our study has some potential limitations. Although smoking and alcohol consumption data are available in HES, it is of questionable quality as the proportion of women reporting either activity is much lower than expected [26, 27]. Residual confounding from BMI, smoking, alcohol consumption, medication, lipid levels and diabetes (since we only adjusted for diabetes recorded in the hospital record) may remain although results from previous studies suggest little further attenuation upon adjustment of some of these variables [28, 29]. Missing data in these variables could not be assumed to be missing at random, which would have allowed a multiple imputation approach. Despite excluding women with a pre-pregnancy hospital record of a diagnosis of chronic hypertension, our analysis may have included women with undiagnosed pre-pregnancy hypertension, which is likely to increase the magnitude of associations. Misclassification between GH and PE may exist, despite our results indicating differential associations with CVD. However, validation studies of HDP in hospital data from Europe, North America and Australia have demonstrated the good reliability and accuracy of GH and PE diagnoses in routinely collected hospital data [30]. Classification of CVD outcomes relied only on hospital admissions, which may exclude less severe presentations of outcomes, including atrial fibrillation and flutter and transient ischemic attack; however, it allowed greater specificity in the CVD subtypes. Furthermore, variation in coding over time or between hospitals may have arisen, likely leading to non-differential misclassification. Finally, individuals who developed a HDP prior to the start of the dataset could not be included, limiting the duration of follow-up to 18 years.

## Conclusions

This study demonstrates that women with prior GH or PE are also at greater risk of common initial manifestations of CVD. Heterogeneous associations were seen across a range of acute and chronic cardiovascular diseases in patients with GH or PE. Further investigations into clinical risk assessment, for example counselling during and/or after an affected pregnancy, and preventive strategies, such as lifestyle changes that limit excess interpregnancy weight gain, are needed.

## Methods

### Study cohort

All female patients in National Health Service hospitals in England with at least one pregnancy ending in a singleton live birth between 1997 and 2015 were identified from the Hospital Episode Statistics (HES) database. Women who had a multiple birth or a hospital record of CVD or chronic hypertension prior to or during the 6 months after delivery were excluded. No restrictions on gestational age at birth were included. This was a conservative approach chosen to mitigate against the circumstance where a woman had an underlying, undiagnosed heart condition prior to pregnancy, which was diagnosed after pregnancy. First delivery was identified by the number of reported previous births recorded in each delivery record. The study was approved by the National Health Service (NHS) Confidentiality Advisory Group; neither informed consent nor ethics committee approval is required for analysis of anonymised routinely collected hospital data.

### Hypertensive disorders of pregnancy

GH and PE diagnoses (including eclampsia and HELLP syndrome) were identified from version 10 of the International Classification of Diseases (ICD-10) codes (O13 for GH, and O14 for PE). Reported cases of either GH or PE were defined as any episode of a hospital admission (obstetric-related or otherwise) with a primary or secondary diagnosis with ICD-10 codes of O13 or O14 recorded between 270 days (9 months) before delivery and 30 days postpartum. Women with both GH and PE diagnoses in the same pregnancy were classified as having a PE diagnosis. Cases of severe PE were identified by ICD-10 codes: O14.1, O14.2, O15.0 and O15.9, and mild/moderate PE were classified using ICD-10 codes: O14.0 and O14.9.

### Cardiovascular outcomes

Primary end points were the initial presentation of CVD occurring after 6 months postpartum. Twenty hierarchical outcomes were identified from ICD-10 codes (Additional file 1: Table S1, Fig. S1): total CVD comprising abdominal aortic aneurysm; coronary heart disease (sub-components: acute myocardial infarction; angina [sub-components: stable and unstable]); atrial fibrillation and flutter; cardiomyopathy [sub-components: dilated and hypertrophic]; heart failure; ventricular arrhythmias, cardiac arrest and sudden cardiac death; peripheral arterial disease; all strokes (sub-components: ischemic stroke; haemorrhagic stroke [subarachnoid haemorrhage; intracerebral haemorrhage]); and transient ischemic attack. Patients were censored at the earliest of the following: the date of their first CVD presentation, the last date in the dataset or the date at which a patient was discharged from hospital due to death.

### Covariates

To aid identification of covariates, direct acyclic graphs (DAG) were produced (Additional file 1: Fig. S2). Available and reliable potential confounders included maternal age at delivery (estimated from the date of delivery and the mother’s birth-month and -year), socioeconomic status (measured by the Index of Multiple Deprivation 2004 categorised into deciles of the scores found in the dataset [31]), ethnicity (defined as White, Black, Asian and other), hospital-recorded diabetes, diagnosed prior to pregnancy, based on in-patient records (identified from ICD-10 codes recorded in the CALIBER research portal [E10-E12, O24.2; https://caliberresearch.org/portal/phenotypes/diabetes]) and year of delivery. A covariate was considered unreliable if it varied across repeated measures in different pregnancies. Covariates were recorded during admission for the birth of the mother’s first infant.

### Statistical analysis

We estimated age- and year-specific incidences of GH and PE and the cumulative incidence of total CVD by year, age and hypertensive status of the first pregnancy. Hazard ratios (HRs) were estimated to quantify the associations between GH and PE and total CVD and pre-specified CVD subtypes using Cox proportional hazards models with time-since-first-delivery as the timescale. We estimated 95% CIs for all comparative groups (including the reference group) that corresponded to the amount of information underlying each group [32]. All models were adjusted for maternal age at delivery, with further adjustment for socioeconomic status, ethnicity and diabetes. The proportional hazards assumption was verified using the test of Grambsch and Therneau [33] and by plotting the Schoenfield residuals.

Primary analyses were restricted to nulliparous women. We further conducted two dose-response secondary analyses amongst multiparous women. First, hazard ratios for the number of pregnancies affected by either GH or PE were calculated (excluding women experiencing GH and PE in separate pregnancies). Second, we calculated HRs for the severity of PE in the 1st pregnancy categorised as severe [ICD-10 codes: O14.1, O14.2, O15.0, O15.9] or mild/moderate [ICD-10 code: O14.0, O14.9]. Sensitivity analyses were conducted, which assessed the impact of starting follow-up at 3 months and 12 months post-partum on analyses of nulliparous women.

Statistical analyses were conducted on Stata v14.1 (Stata Corp., College Station, TX, USA). *P* < 0.05 (2-sided) was considered statistically significant.

## Supplementary Information

**Additional file 1: Table S1**: International Classification of Disease (10th Revision) codes for pre-specified cardiovascular subtypes. **Table S2**: Characteristics of a Woman’s First Pregnancy Resulting in a Singleton Live Birth, England, 1997-2015. **Table S3**: Baseline characteristics of the number of pregnancies affected by gestational hypertension or pre-eclampsia in the study period resulting in a singleton live birth, England, 1997-2015. **Table S4**: Hazard Ratios for risk of pre-specified cardiovascular subtypes more than 6 months after first delivery by history of gestational hypertension compared to women with normotensive pregnancies, England, 1997-2015. **Table S5**: Hazard Ratios for risk of pre-specified cardiovascular subtypes more than 6 months after first delivery by history of pre-eclampsia compared to women with normotensive pregnancies, England, 1997-2015. **Table S6**: Hazard Ratios for risk of pre-specified cardiovascular subtypes more than 6 months after first delivery by the number of pregnancies complicated by gestational hypertension compared to women with normotensive pregnancies, England, 1997-2015. **Table S7**: Hazard Ratios for risk of pre-specified cardiovascular subtypes more than 6 months after first delivery by the number of pregnancies complicated by pre-eclampsia compared to women with normotensive pregnancies, England, 1997-2015. **Table S8**: Hazard Ratios for risk of pre-specified cardiovascular subtypes more than 6 months after first delivery of a pregnancy complicated by mild pre-eclampsia and severe pre-eclampsia compared to women with normotensive pregnancies, England, 1997-2015. **Table S9**: Hazard Ratios for risk of pre-specified cardiovascular subtypes more than 3 and 12 months after first delivery of a pregnancy complicated by pre-eclampsia compared to women with normotensive pregnancies, England, 1997-2015. **Table S10**: Hazard Ratios for risk of pre-specified cardiovascular subtypes more than 3 and 12 months after first delivery of a pregnancy complicated by pre-eclampsia compared to women with normotensive pregnancies, England, 1997-2015. **Figure S1**: Hierarchical diagram showing the relationship between the different pre-specified cardiovascular subtypes. **Figure S2**: Direct acyclic graphs displaying the relationship between A) gestational hypertension and B) pre-eclampsia with cardiovascular disease, confounders and mediators. **Figure S3**: Flow chart of cohort selection. **Figure S4**: Proportions of cardiovascular subtypes more than 6 months after first delivery by history of gestational hypertension or pre-eclampsia compared to women with normotensive pregnancies, England, 1997-2015. **Figure S5**:  Cardiovascular disease risk more than 6 months after first delivery of a pregnancy complicated by gestational hypertension compared to women with normotensive pregnancies over time, England, 1997-2015. **Figure S6**: Cardiovascular disease risk more than 6 months after first delivery of a pregnancy complicated by pre-eclampsia compared to women with normotensive pregnancies over time, England, 1997-2015. **Figure S7**: Hazard Ratios for risk of pre-specified cardiovascular subtypes more than 6 months after first delivery by the number of pregnancies complicated by gestational hypertension compared to women with normotensive pregnancies, England, 1997-2015. **Figure S8**: Hazard Ratios for risk of pre-specified cardiovascular subtypes more than 6 months after first delivery by the number of pregnancies complicated by pre-eclampsia compared to women with normotensive pregnancies, England, 1997-2015. **Figure S9**: Hazard Ratios for risk of pre-specified cardiovascular subtypes more than 6 months after first delivery of a pregnancy complicated by mild pre-eclampsia and severe pre-eclampsia compared to women with normotensive pregnancies, England, 1997-2015.

## Data Availability

Original datasets from HES are available to researchers. Access to data can be obtained via NHS digital (https://digital.nhs.uk/data-and-information/data-tools-and-services/data-services/hospital-episode-statistics/users-uses-and-access-to-hospital-episode-statistics).

## Abbreviations

aHR: Adjusted hazard ratios
BMI: Body mass index
CI: Confidence intervals
CHD: Coronary heart disease
CVD: Cardiovascular disease
DAG: Direct acyclic graphs
GH: Gestational hypertension
HES: Hospital episode statistics
HDP: Hypertensive disorders of pregnancy
ICD: International classification of diseases
NHS: National health service
RR: Relative risks
PE: Pre-eclampsia
