# Supplementary material for: Association between hypertensive disorders of pregnancy and later risk of cardiovascular outcomes
Source: BMC Med. 2022 Jan 25;20:19. doi: 10.1186/s12916-021-02218-8 (PMC8787919; doi:10.1186/s12916-021-02218-8)
Supplement: Supplementary file 1 — Additional file 1: Table S1: International Classification of Disease (10th Revision) codes for pre-specified cardiovascular subtypes. Table S2: Characteristics of a Woman’s First Pregnancy Resulting in a Singleton Live Birth, England, 1997-2015. Table S3: Baseline characteristics of the number of pregnancies affected by gestational hypertension or pre-eclampsia in the study period resulting in a singleton live birth, England, 1997-2015. Table S4: Hazard Ratios for risk of pre-specified cardiovascular subtypes more than 6 months after first delivery by history of gestational hypertension compared to women with normotensive pregnancies, England, 1997-2015. Table S5: Hazard Ratios for risk of pre-specified cardiovascular subtypes more than 6 months after first delivery by history of pre-eclampsia compared to women with normotensive pregnancies, England, 1997-2015. Table S6: Hazard Ratios for risk of pre-specified cardiovascular subtypes more than 6 months after first delivery by the number of pregnancies complicated by gestational hypertension compared to women with normotensive pregnancies, England, 1997-2015. Table S7: Hazard Ratios for risk of pre-specified cardiovascular subtypes more than 6 months after first delivery by the number of pregnancies complicated by pre-eclampsia compared to women with normotensive pregnancies, England, 1997-2015. Table S8: Hazard Ratios for risk of pre-specified cardiovascular subtypes more than 6 months after first delivery of a pregnancy complicated by mild pre-eclampsia and severe pre-eclampsia compared to women with normotensive pregnancies, England, 1997-2015. Table S9: Hazard Ratios for risk of pre-specified cardiovascular subtypes more than 3 and 12 months after first delivery of a pregnancy complicated by pre-eclampsia compared to women with normotensive pregnancies, England, 1997-2015. Table S10: Hazard Ratios for risk of pre-specified cardiovascular subtypes more than 3 and 12 months after first delivery of a pregnancy compl [file 12916_2021_2218_MOESM1_ESM.docx]

# **Association between hypertensive disorders of pregnancy and later risk of cardiovascular outcomes**

Clare Oliver-Williams, PhD; David Stevens, MSc; Rupert A Payne, PhD; Ian B Wilkinson, DM; Gordon CS Smith, MD; Angela Wood, PhD

**Supplementary Material**

eTable 1: International Classification of Disease (10^th^ Revision) codes for pre-specified cardiovascular subtypes

| **Endpoint** | **ICD-10 hospital diagnoses** |
| --- | --- |
| Cardiovascular disease | Includes everything specified elsewhere |
| Coronary heart disease | I11.0, I13.0, I13.2, I20-I24, I25 excluding I25.2, I42, I43, I46, I47.0, I47.2, I48, I50 |
| Angina | I20, I24 |
| Stable angina | I20 excluding I20.0 |
| Unstable angina | I20.0, I24 |
| Acute Myocardial Infarction (MI) | I21-I23 |
| Heart failure | I11.0, I13.0, I13.2, I50 |
| Cardiomyopathy | I42, I43 |
| Dilated cardiomyopathy | I42.0 |
| Hypertrophic Cardiomyopathy | I42.1, I42.2 |
| Atrial Fibrillation and flutter | I48 |
| Ventricular arrhythmias, cardiac arrest and sudden cardiac death | I46, I47.0, I47.2 |
| Transient ischaemic attack | G45.8-G45.9 |
| All Strokes | I60-I64, G46.3-G46.7 |
| Ischaemic stroke | I63 |
| Haemorrhagic Stroke | I60-I62 |
| Subarachnoid haemorrhage | I60 |
| Intracerebral haemorrhage | I61 |
| Peripheral arterial disease | I70.2, I73.9, E11.5, E11.6, E11.62 |
| Abdominal aortic aneurysm | I71.3-I71.9 |

eTable 2: Characteristics of a Woman’s First Pregnancy Resulting in a Singleton Live Birth, England, 1997-2015

|  | All first pregnancies  N=2,359,386 | | Normotensive  n=2,199,567 | | Gestational Hypertension  n=85,277 | | Pre-eclampsia  n=74,542 | |
| --- | --- | --- | --- | --- | --- | --- | --- | --- |
| Person-years | 16,309,307 |  | 15,181,237 |  | 606,769 |  | 521,301 |  |
| Maternal-age at delivery, mean (SD) in years | 26.89 | 5.80 | 26.85 | 5.80 | 27.53 | 5.74 | 27.31 | 5.85 |
| IMD Deciles, n (%) |  |  |  |  |  |  |  |  |
| Least deprived 10% | 192,846 | 8.2% | 178,907 | 8.1% | 7,734 | 9.1% | 6,205 | 8.3% |
| Less deprived 10-20% | 198,731 | 8.4% | 184,487 | 8.4% | 7,754 | 9.1% | 6,490 | 8.7% |
| Less deprived 20-30% | 200,097 | 8.5% | 185,788 | 8.4% | 7,921 | 9.3% | 6,388 | 8.6% |
| Less deprived 30-40% | 203,980 | 8.6% | 189,573 | 8.6% | 7,732 | 9.1% | 6,675 | 9.0% |
| Less deprived 40-50% | 216,683 | 9.2% | 201,701 | 9.2% | 8,091 | 9.5% | 6,891 | 9.2% |
| More deprived 40-50% | 230,349 | 9.8% | 214,569 | 9.8% | 8,449 | 9.9% | 7,331 | 9.8% |
| More deprived 30-40% | 246,749 | 10.5% | 229,899 | 10.5% | 9,029 | 10.6% | 7,821 | 10.5% |
| More deprived 20-30% | 262,672 | 11.1% | 245,083 | 11.1% | 9,440 | 11.1% | 8,149 | 10.9% |
| More deprived 10-20% | 279,334 | 11.8% | 261,765 | 11.9% | 9,168 | 10.8% | 8,401 | 11.3% |
| Most deprived 10% | 311,476 | 13.2% | 292,445 | 13.3% | 9,315 | 10.9% | 9,716 | 13.0% |
| Missing, n (%) | 16,073 | 0.7% | 14,983 | 0.7% | 634 | 0.7% | 456 | 0.6% |
| Ethnicity, n (%) |  |  |  |  |  |  |  |  |
| White | 1,719,750 | 72.9% | 1,598,198 | 72.7% | 65,846 | 77.2% | 55,706 | 74.8% |
| Asian | 247,516 | 10.5% | 234,242 | 10.7% | 6,422 | 7.5% | 6,852 | 9.2% |
| Black | 91,646 | 3.9% | 85,502 | 3.9% | 2,773 | 3.3% | 3,371 | 4.5% |
| Any Other | 74,142 | 3.1% | 70,309 | 3.2% | 1,920 | 2.3% | 1,913 | 2.6% |
| Missing | 225,936 | 9.6% | 210,949 | 9.6% | 8,306 | 9.7% | 6,681 | 9.0% |
| Diabetic, n (%) | 4,709 | 0.2% | 3,886 | 0.2% | 296 | 0.3% | 527 | 0.7% |
| Smoker, n (%) | 50,710 | 2.1% | 47,232 | 2.1% | 1,883 | 2.2% | 1,595 | 2.1% |
| Alcohol Drinker, n (%) | 14,838 | 0.6% | 13,962 | 0.6% | 466 | 0.5% | 410 | 0.6% |
| Subsequent CVD events, n (%) |  |  |  |  |  |  |  |  |
| All CVD | 10806 | 0.5% | 9651 | 0.4% | 583 | 0.7% | 572 | 0.8% |
| Coronary Heart Disease | 2974 | 0.1% | 2621 | 0.1% | 174 | 0.2% | 179 | 0.2% |
| Angina | 1286 | 0.1% | 1142 | 0.1% | 79 | 0.1% | 65 | 0.1% |
| Stable Angina | 749 | 0.0% | 664 | 0.0% | 43 | 0.0% | 42 | 0.0% |
| Unstable Angina | 506 | 0.0% | 448 | 0.0% | 35 | 0.0% | 23 | 0.0% |
| Acute Myocardial Infarction | 453 | 0.0% | 385 | 0.0% | 29 | 0.0% | 39 | 0.0% |
| Heart Failure | 664 | 0.0% | 596 | 0.0% | 36 | 0.0% | 32 | 0.0% |
| Cardiomyopathy | 555 | 0.0% | 468 | 0.0% | 44 | 0.0% | 43 | 0.0% |
| Dilated Cardiomyopathy | 176 | 0.0% | 148 | 0.0% | 16 | 0.0% | 12 | 0.0% |
| Hypertrophic Cardiomyopathy | 72 | 0.0% | 62 | 0.0% | <5 | 0.0% | 8 | 0.0% |
| Atrial Fibrillation and Flutter | 1592 | 0.1% | 1447 | 0.1% | 79 | 0.1% | 66 | 0.1% |
| Ventricular arrhythmias, cardiac arrest and sudden cardiac death | 1037 | 0.0% | 950 | 0.0% | 43 | 0.0% | 44 | 0.0% |
| Transient Ischaemic Attack | 596 | 0.0% | 548 | 0.0% | 19 | 0.0% | 29 | 0.0% |
| All Strokes | 2131 | 0.1% | 1902 | 0.1% | 123 | 0.1% | 106 | 0.1% |
| Ischemic Stroke | 864 | 0.0% | 772 | 0.0% | 47 | 0.0% | 45 | 0.0% |
| Haemorrhagic Stroke | 991 | 0.0% | 885 | 0.0% | 59 | 0.0% | 47 | 0.0% |
| Subarachnoid Haemorrhage | 707 | 0.0% | 633 | 0.0% | 42 | 0.0% | 32 | 0.0% |
| Intracerebral Haemorrhage | 300 | 0.0% | 268 | 0.0% | 17 | 0.0% | 15 | 0.0% |
| Peripheral Arterial Disease | 389 | 0.0% | 355 | 0.0% | 15 | 0.0% | 19 | 0.0% |
| Abdominal Aortic Aneurysm | 58 | 0.0% | 51 | 0.0% | <5 | 0.0% | <5 | 0.0% |

*IMD – Indices of multiple deprivation*

*IMD was categorised into deciles of the scores found in the dataset.*

eTable 3: Baseline characteristics of the number of pregnancies affected by gestational hypertension or pre-eclampsia in the Study Period Resulting in a singleton Live Birth, England, 1997-2015

|  | All first pregnancies | Normotensive | Gestational Hypertension | | Pre-eclampsia | |
| --- | --- | --- | --- | --- | --- | --- |
|  |  |  | 1 | 2+ | 1 | 2+ |
| Total | 4033658 (100%) | 3725949 (100%) | 156979 (100%) | 8132 (100%) | 127580 (100%) | 6122 (100%) |
| Person-years | 16,309,308 | 15006178 | 535329 | 29223.92 | 659736.6 | 38927.27 |
| Mother’s age at delivery | 28.05 (5.74) | 27.99 (5.74) | 28.37 (5.73) | 30.13 (5.19) | 28.73 (5.63) | 30.61 (5.12) |
| Diabetic, n (%) | 9451 (0%) | 7617 (0%) | 959 (1%) | 89 (1%) | 648 (0%) | 48 (1%) |
| Smoker, n (%) | 168669 (4%) | 156126 (4%) | 4750 (4%) | 335 (5%) | 6469 (4%) | 434 (5%) |
| Alcohol Drinker, n (%) | 29701 (1%) | 27683 (1%) | 797 (1%) | 52 (1%) | 1050 (1%) | 54 (1%) |
| IMD Decile |  |  |  |  |  |  |
| Least deprived 10% | 345040 (9%) | 317297 (9%) | 10875 (9%) | 484 (8%) | 14807 (9%) | 771 (9%) |
| Less deprived 10-20% | 345209 (9%) | 317520 (9%) | 11085 (9%) | 521 (9%) | 14457 (9%) | 791 (10%) |
| Less deprived 20-30% | 342136 (8%) | 314890 (8%) | 10814 (8%) | 511 (8%) | 14386 (9%) | 781 (10%) |
| Less deprived 30-40% | 344821 (9%) | 317652 (9%) | 11274 (9%) | 508 (8%) | 13898 (9%) | 777 (10%) |
| Less deprived 40-50% | 362141 (9%) | 334088 (9%) | 11541 (9%) | 486 (8%) | 14507 (9%) | 715 (9%) |
| More deprived 40-50% | 382975 (9%) | 353499 (9%) | 12198 (10%) | 603 (10%) | 15105 (10%) | 737 (9%) |
| More deprived 30-40% | 409131 (10%) | 377700 (10%) | 13019 (10%) | 576 (9%) | 16154 (10%) | 776 (10%) |
| More deprived 20-30% | 438942 (11%) | 406033 (11%) | 13697 (11%) | 691 (11%) | 16792 (11%) | 828 (10%) |
| More deprived 10-20% | 479503 (12%) | 444922 (12%) | 14665 (11%) | 745 (12%) | 17257 (11%) | 897 (11%) |
| Most deprived 10% | 559592 (14%) | 520098 (14%) | 17665 (14%) | 961 (16%) | 18576 (12%) | 1018 (13%) |
| Missing | 23287 (1%) | 21466 (1%) | 705 (1%) | 32 (1%) | 994 (1%) | 40 (0%) |
| Ethnicity |  |  |  |  |  |  |
| White | 2989431 (74%) | 2751733 (74%) | 96398 (76%) | 4530 (74%) | 123081 (78%) | 6650 (82%) |
| Asian | 462011 (11%) | 432944 (12%) | 13169 (10%) | 774 (13%) | 13428 (9%) | 754 (9%) |
| Black | 157242 (4%) | 144955 (4%) | 5997 (5%) | 385 (6%) | 5274 (3%) | 220 (3%) |
| Any Other | 113884 (3%) | 107373 (3%) | 2978 (2%) | 140 (2%) | 3140 (2%) | 115 (1%) |
| Missing | 310209 (8%) | 288160 (8%) | 8996 (7%) | 289 (5%) | 12010 (8%) | 392 (5%) |
| Year of Birth | 1978.66 (6.62) | 1978.71 (6.61) | 1978.29 (6.69) | 1977.65 (6.13) | 1977.92 (6.69) | 1977.05 (6.14) |
| Subsequent CVD Events, n (%) |  |  |  |  |  |  |
| All CVD | 10806 (0%) | 9404 (0%) | 595 (0%) | 50 (1%) | 634 (0%) | 50 (1%) |
| Coronary Heart Disease | 2974 (0%) | 2522 (0%) | 202 (0%) | 18 (0%) | 192 (0%) | 18 (0%) |
| Angina | 1286 (0%) | 1105 (0%) | 76 (0%) | 4 (0%) | 89 (0%) | 5 (0%) |
| Stable Angina | 749 (0%) | 645 (0%) | 46 (0%) | 3 (0%) | 49 (0%) | 3 (0%) |
| Unstable Angina | 506 (0%) | 430 (0%) | 30 (0%) | 1 (0%) | 39 (0%) | 2 (0%) |
| Acute Myocardial Infarction | 453 (0%) | 360 (0%) | 47 (0%) | 5 (0%) | 31 (0%) | 5 (0%) |
| Heart Failure | 664 (0%) | 588 (0%) | 32 (0%) | 3 (0%) | 34 (0%) | 0 (0%) |
| Cardiomyopathy | 555 (0%) | 448 (0%) | 45 (0%) | 3 (0%) | 46 (0%) | 5 (0%) |
| Dilated Cardiomyopathy | 176 (0%) | 137 (0%) | 14 (0%) | 0 (0%) | 17 (0%) | 3 (0%) |
| Hypertrophic Cardiomyopathy | 72 (0%) | 62 (0%) | 7 (0%) | 1 (0%) | 2 (0%) | 0 (0%) |
| Atrial Fibrillation and Flutter | 1592 (0%) | 1414 (0%) | 63 (0%) | 7 (0%) | 99 (0%) | 4 (0%) |
| Ventricular arrhythmias, cardiac arrest and sudden cardiac death | 1037 (0%) | 931 (0%) | 42 (0%) | 2 (0%) | 52 (0%) | 3 (0%) |
| Transient Ischaemic Attack | 596 (0%) | 539 (0%) | 32 (0%) | 3 (0%) | 20 (0%) | 2 (0%) |
| All Strokes | 2131 (0%) | 1866 (0%) | 101 (0%) | 9 (0%) | 123 (0%) | 13 (0%) |
| Ischemic Stroke | 864 (0%) | 757 (0%) | 42 (0%) | 4 (0%) | 44 (0%) | 9 (0%) |
| Haemorrhagic Stroke | 991 (0%) | 867 (0%) | 43 (0%) | 5 (0%) | 62 (0%) | 3 (0%) |
| Subarachnoid Haemorrhage | 707 (0%) | 615 (0%) | 32 (0%) | 3 (0%) | 47 (0%) | 2 (0%) |
| Intracerebral Haemorrhage | 300 (0%) | 268 (0%) | 11 (0%) | 2 (0%) | 15 (0%) | 1 (0%) |
| Peripheral Arterial Disease | 389 (0%) | 349 (0%) | 22 (0%) | 1 (0%) | 15 (0%) | 1 (0%) |
| Abdominal Aortic Aneurysm | 58 (0%) | 50 (0%) | 5 (0%) | 0 (0%) | 1 (0%) | 0 (0%) |

*The values for all first pregnancies do not equal the sum of normotensive, gestational hypertension (1, 2+) and pre-eclampsia (1,2+) because a minority of women had both pre-eclampsia and gestational hypertension and are not included in subsequent analyses.*

eTable 4: Hazard Ratios for risk of pre-specified cardiovascular subtypes more than 6 months after first delivery by history of gestational hypertension compared to women with normotensive pregnancies, England, 1997-2015

|  | All individuals (n=2,284,844) | | Complete Cases (n=2,040,144) | | | |
| --- | --- | --- | --- | --- | --- | --- |
|  | Age Adjusted | |  | Age Adjusted Complete Case | Fully Adjusted |  |
| Disease Subgroup | No. of Cases | HR (95% CI) | No. of Cases | HR (95% CI) | HR (95% CI) |  |
| Overall Cardiovascular Disease | 10806 | 1.47 (1.35, 1.60) | 9653 | 1.44 (1.32, 1.57) | 1.45 (1.33, 1.59) |  |
| Coronary Heart Disease | 2974 | 1.56 (1.34, 1.82) | 2619 | 1.50 (1.27, 1.77) | 1.55 (1.31, 1.83) |  |
| Angina | 1286 | 1.61 (1.29, 2.03) | 1123 | 1.62 (1.27, 2.07) | 1.69 (1.32, 2.16) |  |
| Stable Angina | 749 | 1.52 (1.12, 2.07) | 651 | 1.51 (1.08, 2.11) | 1.56 (1.12, 2.17) |  |
| Unstable Angina | 506 | 1.81 (1.29, 2.56) | 446 | 1.83 (1.27, 2.64) | 1.92 (1.33, 2.77) |  |
| Acute Myocardial Infarction | 453 | 1.71 (1.17, 2.49) | 388 | 1.51 (0.98, 2.33) | 1.49 (0.96, 2.29) |  |
| Heart Failure | 664 | 1.53 (1.09, 2.14) | 605 | 1.64 (1.17, 2.31) | 1.71 (1.21, 2.41) |  |
| Cardiomyopathy | 555 | 2.38 (1.75, 3.25) | 503 | 2.34 (1.69, 3.25) | 2.32 (1.67, 3.22) |  |
| Dilated Cardiomyopathy | 176 | 2.77 (1.65, 4.65) | 161 | 2.84 (1.67, 4.85) | 2.85 (1.67, 4.86) |  |
| Hypertrophic Cardiomyopathy | 72 | 0.81 (0.20, 3.30) | 68 | 0.85 (0.21, 3.49) | 0.81 (0.20, 3.33) |  |
| Atrial Fibrillation and Flutter | 1592 | 1.32 (1.06, 1.66) | 1430 | 1.22 (0.95, 1.56) | 1.20 (0.94, 1.54) |  |
| Ventricular arrhythmias, cardiac arrest and sudden cardiac death | 1037 | 1.15 (0.84, 1.56) | 931 | 1.16 (0.84, 1.61) | 1.16 (0.84, 1.60) |  |
| Transient Ischaemic Attack | 596 | 0.86 (0.54, 1.35) | 540 | 0.90 (0.56, 1.44) | 0.90 (0.56, 1.44) |  |
| All Strokes | 2131 | 1.58 (1.32, 1.90) | 1879 | 1.55 (1.28, 1.89) | 1.56 (1.28, 1.89) |  |
| Ischemic Stroke | 864 | 1.50 (1.11, 2.01) | 764 | 1.60 (1.18, 2.18) | 1.59 (1.17, 2.16) |  |
| Haemorrhagic Stroke | 991 | 1.62 (1.24, 2.11) | 863 | 1.41 (1.04, 1.90) | 1.42 (1.05, 1.92) |  |
| Subarachnoid haemorrhage | 707 | 1.60 (1.17, 2.19) | 612 | 1.40 (0.98, 2.00) | 1.41 (0.99, 2.02) |  |
| Intracerebral haemorrhage | 300 | 1.56 (0.96, 2.55) | 264 | 1.36 (0.78, 2.38) | 1.36 (0.78, 2.37) |  |
| Peripheral arterial disease | 389 | 1.04 (0.62, 1.75) | 360 | 1.05 (0.61, 1.79) | 1.03 (0.60, 1.76) |  |
| Abdominal Aortic aneurysm | 58 | 1.45 (0.45, 4.65) | 55 | 1.54 (0.48, 4.95) | 1.53 (0.48, 4.91) |  |

*Adjusted for maternal age at delivery, socioeconomic status, ethnicity and diabetes*

eTable 5: Hazard Ratios for risk of pre-specified cardiovascular subtypes more than 6 months after first delivery by history of pre-eclampsia compared to women with normotensive pregnancies, England, 1997-2015

|  | All individuals (n=2,274,109) | | Complete Cases (n=2,021,957) | | |
| --- | --- | --- | --- | --- | --- |
|  | Age Adjusted | |  | Age Adjusted | Fully Adjusted |
| Disease Subgroup | No. of Cases | HR (95% CI) | No. of Cases | HR (95% CI) | HR (95% CI) |
| Overall Cardiovascular Disease | 10806 | 1.69 (1.56, 1.84) | 9653 | 1.67 (1.52, 1.82) | 1.62 (1.48, 1.78) |
| Coronary Heart Disease | 2974 | 1.91 (1.64, 2.22) | 2619 | 1.87 (1.59, 2.20) | 1.80 (1.53, 2.12) |
| Angina | 1286 | 1.58 (1.23, 2.03) | 1123 | 1.55 (1.18, 2.03) | 1.52 (1.16, 1.98) |
| Stable Angina | 749 | 1.77 (1.29, 2.41) | 651 | 1.77 (1.27, 2.47) | 1.72 (1.23, 2.40) |
| Unstable Angina | 506 | 1.43 (0.94, 2.17) | 446 | 1.32 (0.83, 2.10) | 1.30 (0.82, 2.07) |
| Acute Myocardial Infarction | 453 | 2.77 (1.99, 3.85) | 388 | 2.70 (1.89, 3.86) | 2.46 (1.72, 3.53) |
| Heart Failure | 664 | 1.56 (1.09, 2.23) | 605 | 1.49 (1.02, 2.18) | 1.44 (0.99, 2.11) |
| Cardiomyopathy | 555 | 2.69 (1.97, 3.68) | 503 | 2.74 (1.98, 3.79) | 2.71 (1.96, 3.74) |
| Dilated Cardiomyopathy | 176 | 2.40 (1.33, 4.32) | 161 | 2.38 (1.29, 4.40) | 2.37 (1.28, 4.38) |
| Hypertrophic Cardiomyopathy | 72 | 3.75 (1.80, 7.84) | 68 | 3.42 (1.56, 7.50) | 3.27 (1.49, 7.19) |
| Atrial Fibrillation and Flutter | 1592 | 1.30 (1.01, 1.66) | 1430 | 1.27 (0.98, 1.66) | 1.25 (0.96, 1.62) |
| Ventricular arrhythmias, cardiac arrest and sudden cardiac death | 1037 | 1.35 (1.00, 1.82) | 931 | 1.32 (0.96, 1.82) | 1.28 (0.93, 1.77) |
| Transient Ischaemic Attack | 596 | 1.53 (1.05, 2.22) | 540 | 1.38 (0.91, 2.08) | 1.35 (0.90, 2.04) |
| All Strokes | 2131 | 1.60 (1.31, 1.94) | 1879 | 1.66 (1.35, 2.04) | 1.64 (1.34, 2.01) |
| Ischemic Stroke | 864 | 1.67 (1.24, 2.26) | 764 | 1.81 (1.33, 2.46) | 1.77 (1.30, 2.42) |
| Haemorrhagic Stroke | 991 | 1.51 (1.13, 2.03) | 863 | 1.49 (1.09, 2.04) | 1.48 (1.08, 2.02) |
| Subarachnoid Haemorrhage | 707 | 1.44 (1.01, 2.05) | 612 | 1.32 (0.89, 1.96) | 1.32 (0.89, 1.95) |
| Intracerebral Haemorrhage | 300 | 1.61 (0.95, 2.70) | 264 | 1.80 (1.07, 3.04) | 1.78 (1.05, 3.00) |
| Peripheral Arterial Disease | 389 | 1.54 (0.97, 2.44) | 360 | 1.29 (0.77, 2.16) | 1.22 (0.73, 2.05) |
| Abdominal Aortic Aneurysm | 58 | 2.25 (0.81, 6.23) | 55 | 2.36 (0.85, 6.56) | 2.36 (0.85, 6.56) |

*Adjusted for maternal age at delivery, socioeconomic status, ethnicity and diabetes*

eTable 6: Hazard Ratios for Risk of pre-specified cardiovascular subtypes more than 6 months after first delivery by the number of pregnancies complicated by gestational hypertension compared to women with normotensive pregnancies, England, 1997-2015

|  | All individuals (n=2,305,607) | | | Complete Cases (n=2,071,776) | | | | |
| --- | --- | --- | --- | --- | --- | --- | --- | --- |
|  |  | Age Adjusted | |  | Age Adjusted | | Fully Adjusted | |
| Disease Subgroup | No. of Cases | 1 GH vs Null | 2 GH vs Null | No. of Cases | 1 GH vs Null | 2 GH vs Null | 1 GH vs Null | 2 GH vs Null |
| Overall Cardiovascular Disease | 10793 | 1.49 (1.38, 1.61) | 1.80 (1.36, 2.37) | 9658 | 1.46 (1.34, 1.58) | 1.66 (1.23, 2.23) | 1.46 (1.34, 1.58) | 1.64 (1.21, 2.21) |
| Coronary Heart Disease | 2972 | 1.62 (1.41, 1.87) | 2.15 (1.35, 3.41) | 2624 | 1.61 (1.39, 1.87) | 1.93 (1.16, 3.20) | 1.63 (1.41, 1.90) | 1.93 (1.16, 3.20) |
| Angina | 1284 | 1.71 (1.39, 2.10) | 1.41 (0.58, 3.38) | 1128 | 1.74 (1.40, 2.17) | 1.50 (0.63, 3.62) | 1.79 (1.44, 2.22) | 1.54 (0.64, 3.71) |
| Stable Angina | 748 | 1.62 (1.22, 2.14) | - | 654 | 1.61 (1.20, 2.17) | - | 1.64 (1.22, 2.21) | - |
| Unstable Angina | 505 | 1.92 (1.40, 2.63) | - | 447 | 2.00 (1.44, 2.77) | - | 2.07 (1.49, 2.87) | - |
| Acute Myocardial Infarction | 453 | 1.78 (1.26, 2.53) | 3.99 (1.65, 9.61) | 388 | 1.81 (1.24, 2.63) | - | 1.76 (1.21, 2.56) | - |
| Heart Failure | 662 | 1.33 (0.95, 1.87) | - | 604 | 1.42 (1.00, 2.00) | - | 1.46 (1.03, 2.07) | - |
| Cardiomyopathy | 554 | 2.35 (1.77, 3.14) | 4.22 (1.75, 10.18) | 503 | 2.31 (1.70, 3.12) | - | 2.28 (1.68, 3.09) | - |
| Dilated Cardiomyopathy | 175 | 2.82 (1.70, 4.68) | - | 160 | 2.89 (1.72, 4.87) | - | 2.90 (1.72, 4.89) | - |
| Hypertrophic Cardiomyopathy | 72 | - | - | 68 | - | - | - | - |
| Atrial Fibrillation and Flutter | 1592 | 1.53 (1.26, 1.87) | - | 1431 | 1.41 (1.13, 1.75) | - | 1.39 (1.12, 1.72) | - |
| Ventricular arrhythmias, cardiac arrest and sudden cardiac death | 1035 | 1.28 (0.98, 1.68) | - | 932 | 1.31 (0.99, 1.74) | - | 1.29 (0.97, 1.71) | - |
| Transient Ischaemic Attack | 596 | 0.83 (0.53, 1.29) | - | 541 | 0.86 (0.55, 1.36) | - | 0.86 (0.55, 1.36) | - |
| All Strokes | 2126 | 1.46 (1.22, 1.74) | 2.38 (1.38, 4.10) | 1878 | 1.43 (1.19, 1.73) | 2.18 (1.21, 3.95) | 1.42 (1.18, 1.72) | 2.16 (1.19, 3.90) |
| Ischemic Stroke | 863 | 1.29 (0.96, 1.73) | 3.94 (2.04, 7.60) | 763 | 1.36 (1.00, 1.85) | 3.81 (1.90, 7.64) | 1.34 (0.99, 1.82) | 3.72 (1.85, 7.45) |
| Haemorrhagic Stroke | 987 | 1.58 (1.23, 2.02) | - | 863 | 1.41 (1.07, 1.86) | - | 1.41 (1.07, 1.86) | - |
| Subarachnoid Haemorrhage | 704 | 1.66 (1.25, 2.22) | - | 614 | 1.48 (1.08, 2.05) | - | 1.49 (1.08, 2.05) | - |
| Intracerebral Haemorrhage | 299 | 1.27 (0.77, 2.11) | - | 262 | 1.15 (0.65, 2.06) | - | 1.14 (0.64, 2.04) | - |
| Peripheral Arterial Disease | 389 | 0.96 (0.58, 1.59) | - | 360 | 0.96 (0.57, 1.62) | - | 0.93 (0.55, 1.57) | - |
| Abdominal Aortic Aneurysm | 58 | - | - | 56 | - | - | - | - |

*GH – gestational hypertension. Adjusted for maternal age at delivery, socioeconomic status, ethnicity and diabetes*

eTable 7: Hazard Ratios for risk of pre-specified cardiovascular subtypes more than 6 months after first delivery by the number of pregnancies complicated by pre-eclampsia compared to women with normotensive pregnancies, England, 1997-2015

|  | All individuals (n=2,141,376) | | | Complete Cases (n=1,907,637) | | | | |
| --- | --- | --- | --- | --- | --- | --- | --- | --- |
|  |  | Age Adjusted | |  | Age Adjusted | | Fully Adjusted | |
| Disease Subgroup | No. of Cases | 1 PE vs Null | 2 PE vs Null | No. of Cases | 1 PE vs Null | 2 PE vs Null | 1 PE vs Null | 2 PE vs Null |
| Overall Cardiovascular Disease | 10793 | 1.74 (1.61, 1.88) | 2.46 (1.86, 3.24) | 9658 | 1.71 (1.57, 1.86) | 2.36 (1.76, 3.16) | 1.65 (1.51, 1.79) | 2.23 (1.67, 2.99) |
| Coronary Heart Disease | 2972 | 2.16 (1.88, 2.48) | 3.03 (1.91, 4.81) | 2624 | 2.08 (1.80, 2.42) | 3.06 (1.90, 4.93) | 1.98 (1.70, 2.29) | 2.81 (1.75, 4.53) |
| Angina | 1284 | 1.85 (1.48, 2.31) | - | 1128 | 1.75 (1.37, 2.24) | - | 1.70 (1.33, 2.16) | - |
| Stable Angina | 748 | 1.92 (1.44, 2.56) | - | 654 | 1.84 (1.35, 2.52) | - | 1.76 (1.29, 2.41) | - |
| Unstable Angina | 505 | 1.87 (1.31, 2.67) | - | 447 | 1.74 (1.18, 2.58) | - | 1.70 (1.15, 2.52) | - |
| Acute Myocardial Infarction | 453 | 3.46 (2.61, 4.60) | 5.79 (2.40, 13.94) | 388 | 3.49 (2.57, 4.73) | - | 3.16 (2.33, 4.29) | - |
| Heart Failure | 662 | 1.52 (1.07, 2.16) | - | 604 | 1.44 (0.99, 2.10) | - | 1.39 (0.96, 2.03) | - |
| Cardiomyopathy | 554 | 2.82 (2.11, 3.77) | - | 503 | 2.86 (2.12, 3.87) | - | 2.82 (2.09, 3.81) | - |
| Dilated Cardiomyopathy | 175 | 2.89 (1.67, 5.01) | - | 160 | 2.90 (1.64, 5.13) | - | 2.89 (1.63, 5.12) | - |
| Hypertrophic Cardiomyopathy | 72 | 3.15 (1.44, 6.89) | - | 68 | 3.27 (1.49, 7.16) | - | 3.06 (1.39, 6.73) | - |
| Atrial Fibrillation and Flutter | 1592 | 1.22 (0.95, 1.56) | 2.25 (1.07, 4.72) | 1431 | 1.25 (0.97, 1.62) | 1.71 (0.71, 4.12) | 1.23 (0.95, 1.58) | 1.66 (0.69, 4.01) |
| Ventricular arrhythmias, cardiac arrest and sudden cardiac death | 1035 | 1.26 (0.93, 1.71) | - | 932 | 1.22 (0.89, 1.69) | - | 1.18 (0.85, 1.63) | - |
| Transient Ischaemic Attack | 596 | 1.64 (1.15, 2.34) | - | 541 | 1.51 (1.03, 2.21) | - | 1.48 (1.01, 2.18) | - |
| All Strokes | 2126 | 1.49 (1.23, 1.81) | 2.24 (1.17, 4.31) | 1878 | 1.52 (1.24, 1.87) | 2.43 (1.26, 4.68) | 1.49 (1.22, 1.83) | 2.35 (1.22, 4.53) |
| Ischemic Stroke | 863 | 1.53 (1.13, 2.07) | - | 763 | 1.56 (1.14, 2.14) | - | 1.51 (1.10, 2.08) | - |
| Haemorrhagic Stroke | 987 | 1.36 (1.01, 1.84) | 2.74 (1.14, 6.60) | 863 | 1.35 (0.98, 1.85) | 2.98 (1.24, 7.17) | 1.34 (0.97, 1.83) | 2.93 (1.22, 7.05) |
| Subarachnoid Haemorrhage | 704 | 1.43 (1.01, 2.02) | - | 614 | 1.35 (0.92, 1.96) | - | 1.34 (0.92, 1.95) | - |
| Intracerebral Haemorrhage | 299 | 1.14 (0.63, 2.05) | - | 262 | 1.28 (0.70, 2.35) | - | 1.25 (0.68, 2.29) | - |
| Peripheral Arterial Disease | 389 | 1.74 (1.15, 2.65) | - | 360 | 1.51 (0.95, 2.39) | - | 1.40 (0.88, 2.22) | - |
| Abdominal Aortic Aneurysm | 58 | 2.76 (1.10, 6.93) | - | 56 | 2.84 (1.13, 7.13) | - | 2.85 (1.13, 7.15) | - |

*PE – pre-eclampsia. Adjusted for maternal age at delivery, socioeconomic status, ethnicity and diabetes*

eTable 8: Hazard Ratios for risk of pre-specified cardiovascular subtypes more than 6 months after first delivery of a pregnancy complicated by mild pre-eclampsia and severe pre-eclampsia compared to women with normotensive pregnancies, England, 1997-2015

|  | All individuals (n=1,819,804) | | | Complete Cases (n=1,567,652) | | | | | |
| --- | --- | --- | --- | --- | --- | --- | --- | --- | --- |
|  |  | Age Adjusted | |  | Age Adjusted | | Fully Adjusted | | |
| Disease Subgroup | No. of Cases | Mild PE vs Null | Severe PE vs Null | No. of Cases | Mild PE vs Null | Severe PE vs Null | Mild PE vs Null | Severe PE vs Null | P-value for heterogeneity |
| Overall Cardiovascular Disease | 10175 | 1.64 (1.49, 1.80) | 1.99 (1.66, 2.40) | 9104 | 1.61 (1.45, 1.77) | 1.98 (1.63, 2.41) | 1.57 (1.42, 1.73) | 1.91 (1.57, 2.32) | <0.001 |
| Coronary Heart Disease | 2789 | 2.00 (1.70, 2.35) | 1.47 (0.98, 2.21) | 2463 | 1.96 (1.65, 2.32) | 1.46 (0.94, 2.26) | 1.90 (1.60, 2.25) | 1.36 (0.88, 2.11) | <0.001 |
| Angina | 1200 | 1.56 (1.18, 2.05) | 1.16 (0.58, 2.32) | 1048 | 1.53 (1.14, 2.05) | 1.17 (0.56, 2.46) | 1.51 (1.12, 2.03) | 1.11 (0.53, 2.33) | 0.057 |
| Stable Angina | 701 | 1.80 (1.29, 2.52) | 1.26 (0.52, 3.02) | 610 | 1.81 (1.27, 2.59) | - | 1.77 (1.24, 2.53) | - | 0.02 |
| Unstable Angina | 469 | 1.31 (0.82, 2.11) | - | 413 | 1.21 (0.72, 2.05) | - | 1.21 (0.72, 2.05) | - | 0.893 |
| Acute Myocardial Infarction | 422 | 2.94 (2.09, 4.13) | 2.14 (0.89, 5.15) | 365 | 2.86 (1.97, 4.14) | - | 2.58 (1.78, 3.74) | - | <0.001 |
| Heart Failure | 626 | 1.33 (0.88, 2.03) | 2.54 (1.32, 4.88) | 568 | 1.25 (0.80, 1.97) | 2.50 (1.25, 5.00) | 1.23 (0.78, 1.92) | 2.29 (1.15, 4.58) | 0.098 |
| Cardiomyopathy | 509 | 2.35 (1.64, 3.35) | 4.79 (2.78, 8.26) | 463 | 2.39 (1.65, 3.46) | 4.88 (2.77, 8.59) | 2.35 (1.62, 3.40) | 4.83 (2.74, 8.51) | <0.001 |
| Dilated Cardiomyopathy | 160 | 2.27 (1.18, 4.37) | - | 146 | 2.19 (1.09, 4.37) | - | 2.18 (1.09, 4.36) | - | 0.001 |
| Hypertrophic Cardiomyopathy | 70 | 2.88 (1.20, 6.92) | - | 66 | - | - | - | - | 0.001 |
| Atrial Fibrillation and Flutter | 1505 | 1.26 (0.95, 1.65) | 1.40 (0.79, 2.46) | 1356 | 1.25 (0.93, 1.66) | 1.29 (0.70, 2.41) | 1.22 (0.91, 1.63) | 1.27 (0.68, 2.37) | 0.495 |
| Ventricular arrhythmias, cardiac arrest and sudden cardiac death | 992 | 1.22 (0.86, 1.72) | 1.79 (0.96, 3.32) | 890 | 1.13 (0.78, 1.65) | 1.99 (1.07, 3.70) | 1.10 (0.76, 1.61) | 1.92 (1.04, 3.58) | 0.209 |
| Transient Ischaemic Attack | 575 | 1.71 (1.16, 2.51) | - | 520 | 1.65 (1.10, 2.49) | - | 1.64 (1.09, 2.47) | - | 0.116 |
| All Strokes | 1996 | 1.49 (1.19, 1.86) | 2.23 (1.51, 3.30) | 1765 | 1.56 (1.24, 1.96) | 2.23 (1.47, 3.39) | 1.54 (1.23, 1.94) | 2.20 (1.45, 3.34) | <0.001 |
| Ischemic Stroke | 814 | 1.43 (1.01, 2.04) | 2.63 (1.49, 4.63) | 717 | 1.62 (1.14, 2.30) | 2.51 (1.35, 4.67) | 1.59 (1.11, 2.25) | 2.47 (1.33, 4.59) | 0.002 |
| Haemorrhagic Stroke | 924 | 1.50 (1.09, 2.07) | 1.91 (1.03, 3.55) | 813 | 1.45 (1.03, 2.05) | 1.96 (1.02, 3.76) | 1.44 (1.02, 2.04) | 1.93 (1.00, 3.71) | 0.042 |
| Subarachnoid Haemorrhage | 658 | 1.53 (1.05, 2.24) | 1.35 (0.56, 3.24) | 575 | 1.41 (0.93, 2.14) | - | 1.41 (0.93, 2.13) | - | 0.44 |
| Intracerebral Haemorrhage | 281 | 1.33 (0.72, 2.47) | 3.10 (1.29, 7.45) | 250 | 1.49 (0.80, 2.77) | 3.54 (1.47, 8.50) | 1.46 (0.79, 2.72) | 3.47 (1.44, 8.34) | 0.027 |
| Peripheral Arterial Disease | 369 | 1.12 (0.62, 2.01) | 3.38 (1.61, 7.09) | 341 | 0.86 (0.43, 1.72) | 3.10 (1.39, 6.91) | 0.81 (0.41, 1.62) | 2.92 (1.31, 6.51) | 0.065 |

*PE – pre-eclampsia. Adjusted for maternal age at delivery, socioeconomic status, ethnicity and diabetes. Abdominal Aortic Aneurysm was not included as there were too few cases*

eTable 9: Hazard Ratios for risk of pre-specified cardiovascular subtypes more than 3 and 12 months after first delivery of a pregnancy complicated by pre-eclampsia compared to women with normotensive pregnancies, England, 1997-2015

| **Disease Subgroup** | **Follow-up time beginning at 3 months postpartum** | | | | | **Follow-up time beginning at 12 months postpartum** | | | | |
| --- | --- | --- | --- | --- | --- | --- | --- | --- | --- | --- |
|  | **All individuals** | | **Complete Cases** | | | **All individuals** | | **Complete Cases** | | |
|  | **Age Adjusted** | |  | **Age Adjusted** | **Fully Adjusted** | **Age Adjusted** | |  | **Age Adjusted** | **Fully Adjusted** |
|  | **No. of Cases** | **HR (95% CI)** | **No. of Cases** | **HR (95% CI)** | **HR (95% CI)** | **No. of Cases** | **HR (95% CI)** | **No. of Cases** | **HR (95% CI)** | **HR (95% CI)** |
| **Overall Cardiovascular Disease** | 10957 | 1.48 (1.36, 1.60) | 9797 | 1.44 (1.32, 1.58) | 1.46 (1.33, 1.59) | 10524 | 1.46 (1.34, 1.59) | 9391 | 1.42 (1.30, 1.56) | 1.43 (1.31, 1.57) |
| **Coronary Heart Disease** | 3005 | 1.55 (1.33, 1.80) | 2650 | 1.49 (1.26, 1.75) | 1.53 (1.30, 1.81) | 2931 | 1.55 (1.33, 1.81) | 2582 | 1.49 (1.26, 1.76) | 1.54 (1.30, 1.82) |
| **Angina** | 1300 | 1.60 (1.27, 2.01) | 1137 | 1.60 (1.26, 2.05) | 1.67 (1.31, 2.13) | 1268 | 1.62 (1.28, 2.03) | 1106 | 1.62 (1.27, 2.07) | 1.69 (1.32, 2.16) |
| **Stable Angina** | 757 | 1.50 (1.10, 2.05) | 659 | 1.49 (1.07, 2.08) | 1.54 (1.10, 2.15) | 737 | 1.54 (1.13, 2.10) | 640 | 1.54 (1.10, 2.14) | 1.59 (1.14, 2.22) |
| **Unstable Angina** | 512 | 1.79 (1.27, 2.53) | 452 | 1.81 (1.25, 2.61) | 1.89 (1.31, 2.73) | 500 | 1.78 (1.25, 2.52) | 440 | 1.79 (1.23, 2.59) | 1.88 (1.29, 2.72) |
| **Acute Myocardial Infarction** | 459 | 1.69 (1.16, 2.46) | 394 | 1.49 (0.97, 2.29) | 1.47 (0.95, 2.26) | 447 | 1.67 (1.14, 2.45) | 383 | 1.45 (0.94, 2.26) | 1.43 (0.92, 2.23) |
| **Heart Failure** | 679 | 1.58 (1.14, 2.19) | 619 | 1.70 (1.22, 2.37) | 1.77 (1.27, 2.47) | 645 | 1.53 (1.08, 2.15) | 587 | 1.64 (1.16, 2.32) | 1.71 (1.20, 2.41) |
| **Cardiomyopathy** | 574 | 2.46 (1.83, 3.33) | 521 | 2.44 (1.78, 3.35) | 2.42 (1.76, 3.32) | 541 | 2.33 (1.70, 3.19) | 489 | 2.28 (1.63, 3.19) | 2.26 (1.61, 3.16) |
| **Dilated Cardiomyopathy** | 185 | 2.98 (1.83, 4.86) | 170 | 3.06 (1.85, 5.07) | 3.08 (1.86, 5.10) | 169 | 2.50 (1.44, 4.32) | 154 | 2.54 (1.44, 4.50) | 2.55 (1.44, 4.51) |
| **Hypertrophic Cardiomyopathy** | 73 | 0.80 (0.19, 3.25) | 69 | 0.84 (0.20, 3.43) | 0.80 (0.20, 3.28) | 72 | 0.81 (0.20, 3.30) | 68 | 0.85 (0.21, 3.49) | 0.81 (0.20, 3.33) |
| **Atrial Fibrillation and Flutter** | 1612 | 1.31 (1.04, 1.64) | 1448 | 1.20 (0.94, 1.54) | 1.19 (0.93, 1.52) | 1542 | 1.28 (1.01, 1.61) | 1382 | 1.16 (0.89, 1.50) | 1.14 (0.88, 1.48) |
| **Ventricular arrhythmias, cardiac arrest and sudden cardiac death** | 1054 | 1.13 (0.83, 1.53) | 947 | 1.15 (0.83, 1.58) | 1.14 (0.83, 1.57) | 1007 | 1.13 (0.82, 1.54) | 903 | 1.17 (0.85, 1.62) | 1.17 (0.84, 1.62) |
| **Transient Ischaemic Attack** | 602 | 0.85 (0.54, 1.34) | 546 | 0.89 (0.55, 1.42) | 0.89 (0.56, 1.42) | 578 | 0.88 (0.56, 1.40) | 524 | 0.93 (0.58, 1.48) | 0.93 (0.58, 1.49) |
| **All Strokes** | 2159 | 1.61 (1.34, 1.92) | 1905 | 1.58 (1.30, 1.92) | 1.58 (1.30, 1.92) | 2063 | 1.56 (1.30, 1.88) | 1815 | 1.53 (1.25, 1.87) | 1.53 (1.25, 1.87) |
| **Ischemic Stroke** | 879 | 1.54 (1.15, 2.06) | 778 | 1.65 (1.23, 2.23) | 1.64 (1.22, 2.21) | 841 | 1.50 (1.11, 2.02) | 743 | 1.60 (1.18, 2.18) | 1.59 (1.17, 2.17) |
| **Haemorrhagic Stroke** | 1002 | 1.60 (1.23, 2.08) | 873 | 1.39 (1.03, 1.88) | 1.40 (1.04, 1.90) | 954 | 1.56 (1.19, 2.05) | 828 | 1.33 (0.97, 1.82) | 1.34 (0.98, 1.84) |
| **Subarachnoid Haemorrhage** | 714 | 1.59 (1.16, 2.17) | 619 | 1.38 (0.97, 1.98) | 1.40 (0.98, 2.00) | 685 | 1.57 (1.14, 2.16) | 592 | 1.35 (0.93, 1.95) | 1.37 (0.95, 1.98) |
| **Intracerebral Haemorrhage** | 304 | 1.54 (0.94, 2.51) | 267 | 1.34 (0.77, 2.35) | 1.34 (0.77, 2.34) | 282 | 1.45 (0.86, 2.45) | 246 | 1.22 (0.67, 2.24) | 1.22 (0.67, 2.24) |
| **Peripheral Arterial Disease** | 392 | 1.04 (0.62, 1.74) | 363 | 1.04 (0.61, 1.77) | 1.02 (0.60, 1.74) | 376 | 1.08 (0.64, 1.81) | 347 | 1.08 (0.63, 1.85) | 1.07 (0.62, 1.83) |
| **Abdominal Aortic Aneurysm** | 59 | 1.42 (0.44, 4.56) | 56 | 1.51 (0.47, 4.85) | 1.49 (0.46, 4.80) | 57 | 1.47 (0.46, 4.73) | 54 | 1.57 (0.49, 5.04) | 1.56 (0.48, 5.01) |

*Adjusted for maternal age at delivery, socioeconomic status, ethnicity and diabetes*

eTable 10: Hazard Ratios for risk of pre-specified cardiovascular subtypes more than 3 and 12 months after first delivery of a pregnancy complicated by pre-eclampsia compared to women with normotensive pregnancies, England, 1997-2015

| **Disease Subgroup** | **Follow-up time beginning at 3 months postpartum** | | | | | **Follow-up time beginning at 12 months postpartum** | | | | |
| --- | --- | --- | --- | --- | --- | --- | --- | --- | --- | --- |
|  | **All individuals** | | **Complete Cases** | | | **All individuals** | | **Complete Cases** | | |
|  | **Age Adjusted** | |  | **Age Adjusted** | **Fully Adjusted** | **Age Adjusted** | |  | **Age Adjusted** | **Fully Adjusted** |
|  | **No. of Cases** | **HR (95% CI)** | **No. of Cases** | **HR (95% CI)** | **HR (95% CI)** | **No. of Cases** | **HR (95% CI)** | **No. of Cases** | **HR (95% CI)** | **HR (95% CI)** |
| **Overall Cardiovascular Disease** | 10957 | 1.70 (1.57, 1.85) | 9797 | 1.67 (1.53, 1.83) | 1.63 (1.49, 1.78) | 10524 | 1.70 (1.56, 1.85) | 9391 | 1.67 (1.52, 1.82) | 1.62 (1.48, 1.78) |
| **Coronary Heart Disease** | 3005 | 1.91 (1.64, 2.22) | 2650 | 1.87 (1.59, 2.20) | 1.81 (1.54, 2.12) | 2931 | 1.92 (1.65, 2.24) | 2582 | 1.88 (1.60, 2.21) | 1.81 (1.54, 2.13) |
| **Angina** | 1300 | 1.57 (1.22, 2.01) | 1137 | 1.53 (1.17, 2.00) | 1.50 (1.14, 1.96) | 1268 | 1.61 (1.25, 2.06) | 1106 | 1.57 (1.20, 2.06) | 1.54 (1.18, 2.02) |
| **Stable Angina** | 757 | 1.75 (1.28, 2.39) | 659 | 1.75 (1.26, 2.44) | 1.70 (1.22, 2.37) | 737 | 1.79 (1.31, 2.45) | 640 | 1.80 (1.29, 2.52) | 1.75 (1.26, 2.45) |
| **Unstable Angina** | 512 | 1.41 (0.93, 2.14) | 452 | 1.31 (0.82, 2.07) | 1.29 (0.81, 2.04) | 500 | 1.44 (0.95, 2.19) | 440 | 1.34 (0.84, 2.12) | 1.32 (0.83, 2.09) |
| **Acute Myocardial Infarction** | 459 | 2.81 (2.03, 3.89) | 394 | 2.74 (1.92, 3.90) | 2.50 (1.76, 3.57) | 447 | 2.80 (2.02, 3.90) | 383 | 2.72 (1.90, 3.89) | 2.48 (1.73, 3.56) |
| **Heart Failure** | 679 | 1.53 (1.07, 2.18) | 619 | 1.46 (1.00, 2.13) | 1.42 (0.97, 2.07) | 645 | 1.50 (1.04, 2.17) | 587 | 1.42 (0.96, 2.10) | 1.38 (0.93, 2.04) |
| **Cardiomyopathy** | 574 | 2.67 (1.96, 3.63) | 521 | 2.72 (1.97, 3.74) | 2.69 (1.95, 3.70) | 541 | 2.75 (2.01, 3.77) | 489 | 2.81 (2.03, 3.89) | 2.78 (2.01, 3.85) |
| **Dilated Cardiomyopathy** | 185 | 2.29 (1.27, 4.13) | 170 | 2.27 (1.23, 4.19) | 2.25 (1.22, 4.17) | 169 | 2.47 (1.37, 4.46) | 154 | 2.46 (1.33, 4.55) | 2.45 (1.33, 4.54) |
| **Hypertrophic Cardiomyopathy** | 73 | 3.70 (1.77, 7.72) | 69 | 3.37 (1.54, 7.37) | 3.22 (1.47, 7.08) | 72 | 3.75 (1.80, 7.84) | 68 | 3.42 (1.56, 7.49) | 3.27 (1.49, 7.18) |
| **Atrial Fibrillation and Flutter** | 1612 | 1.34 (1.05, 1.71) | 1448 | 1.33 (1.03, 1.71) | 1.30 (1.01, 1.68) | 1542 | 1.31 (1.02, 1.69) | 1382 | 1.29 (0.99, 1.68) | 1.27 (0.97, 1.65) |
| **Ventricular arrhythmias, cardiac arrest and sudden cardiac death** | 1054 | 1.36 (1.01, 1.84) | 947 | 1.34 (0.97, 1.84) | 1.30 (0.95, 1.79) | 1007 | 1.39 (1.02, 1.88) | 903 | 1.36 (0.99, 1.87) | 1.32 (0.96, 1.82) |
| **Transient Ischaemic Attack** | 602 | 1.51 (1.04, 2.19) | 546 | 1.36 (0.90, 2.05) | 1.34 (0.89, 2.02) | 578 | 1.52 (1.04, 2.23) | 524 | 1.36 (0.90, 2.07) | 1.34 (0.88, 2.03) |
| **All Strokes** | 2159 | 1.63 (1.34, 1.97) | 1905 | 1.67 (1.37, 2.05) | 1.66 (1.35, 2.03) | 2063 | 1.64 (1.35, 2.00) | 1815 | 1.71 (1.40, 2.10) | 1.69 (1.38, 2.08) |
| **Ischemic Stroke** | 879 | 1.76 (1.32, 2.36) | 778 | 1.86 (1.38, 2.52) | 1.83 (1.35, 2.48) | 841 | 1.71 (1.26, 2.31) | 743 | 1.85 (1.36, 2.51) | 1.81 (1.33, 2.47) |
| **Haemorrhagic Stroke** | 1002 | 1.50 (1.12, 2.01) | 873 | 1.47 (1.08, 2.02) | 1.46 (1.07, 2.00) | 954 | 1.57 (1.17, 2.10) | 828 | 1.54 (1.13, 2.12) | 1.53 (1.12, 2.10) |
| **Subarachnoid Haemorrhage** | 714 | 1.42 (1.00, 2.03) | 619 | 1.31 (0.88, 1.94) | 1.30 (0.88, 1.93) | 685 | 1.48 (1.04, 2.11) | 592 | 1.36 (0.92, 2.02) | 1.36 (0.92, 2.01) |
| **Intracerebral Haemorrhage** | 304 | 1.58 (0.94, 2.66) | 267 | 1.78 (1.06, 3.00) | 1.76 (1.04, 2.96) | 282 | 1.70 (1.01, 2.86) | 246 | 1.92 (1.14, 3.24) | 1.89 (1.12, 3.19) |
| **Peripheral Arterial Disease** | 392 | 1.53 (0.96, 2.42) | 363 | 1.28 (0.76, 2.14) | 1.21 (0.72, 2.03) | 376 | 1.50 (0.94, 2.42) | 347 | 1.24 (0.73, 2.12) | 1.18 (0.69, 2.01) |
| **Abdominal Aortic Aneurysm** | 59 | 2.21 (0.80, 6.12) | 56 | 2.32 (0.84, 6.43) | 2.32 (0.84, 6.43) | 57 | 2.29 (0.83, 6.35) | 54 | 2.41 (0.87, 6.69) | 2.41 (0.87, 6.69) |

*Adjusted for maternal age at delivery, socioeconomic status, ethnicity and diabetes*

eFigure 1: Hierarchical diagram showing the relationship between the different pre-specified cardiovascular subtypes.

Cardiovascular Disease

Coronary Heart Disease

All Strokes

Angina

Transient Ischemic Attack

Acute Myocardial Infarction

Atrial Fibrillation and Flutter

Heart Failure

Ventricular arrhythmias, cardiac arrest & sudden cardiac death

Cardiomyopathy

Dilated Cardiomyopathy

Hypertrophic Cardiomyopathy

Stable Angina

Unstable Angina

Haemorrhagic Stroke

Ischemic Stroke

Intracerebral haemorrhage

Subarachnoid haemorrhage

Peripheral Arterial Disease

Abdominal Aortic Aneurysm

eFigure 2: Direct acyclic graphs displaying the relatipnship between A) gestational hypertension and B) pre-eclampsia with cardiovascular disease, confounders and mediators


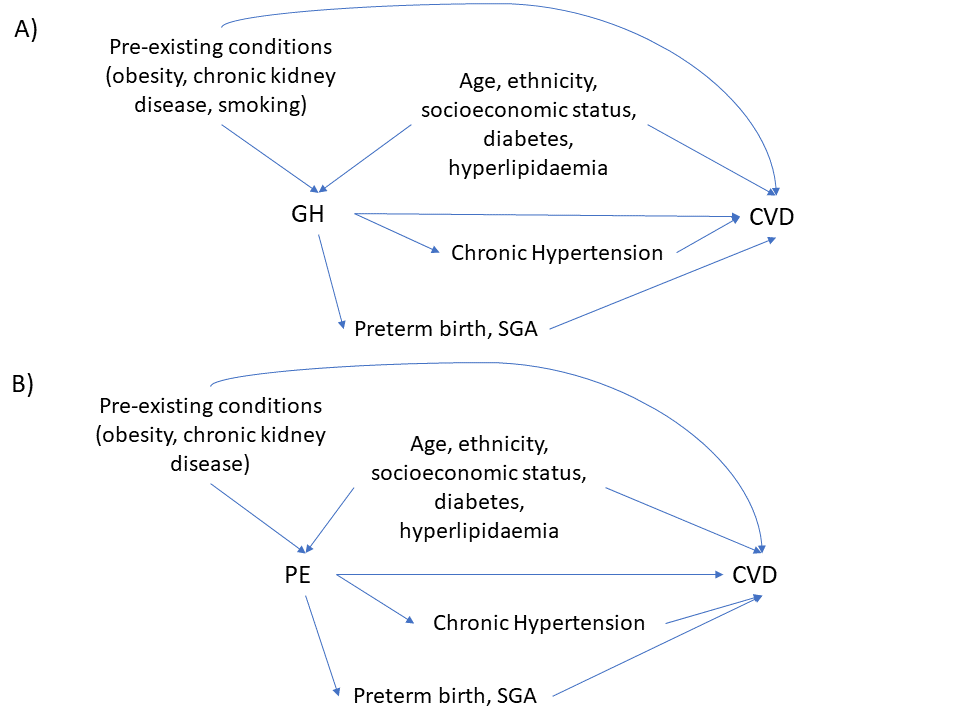


*CVD – cardiovascular disease, GH – gestational hypertension, PE – pre-eclampsia, SGA – small for gestational age infant*

eFigure 3: Flow chart of cohort selection


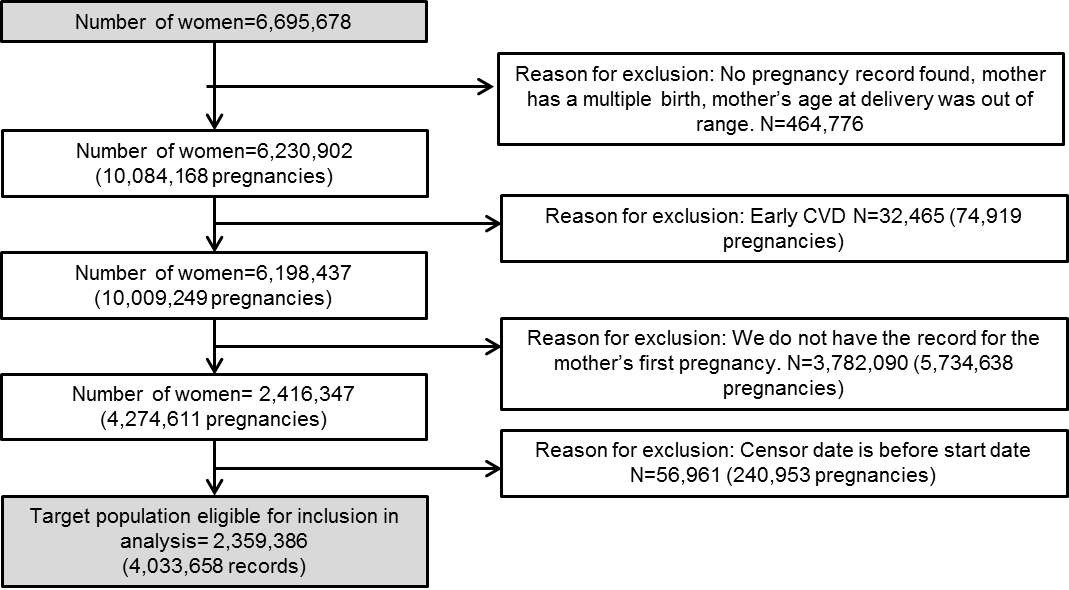


eFigure 4: Proportions of cardiovascular subtypes more than 6 months after first delivery by history of gestational hypertension or pre-eclampsia compared to women with normotensive pregnancies, England, 1997-2015.


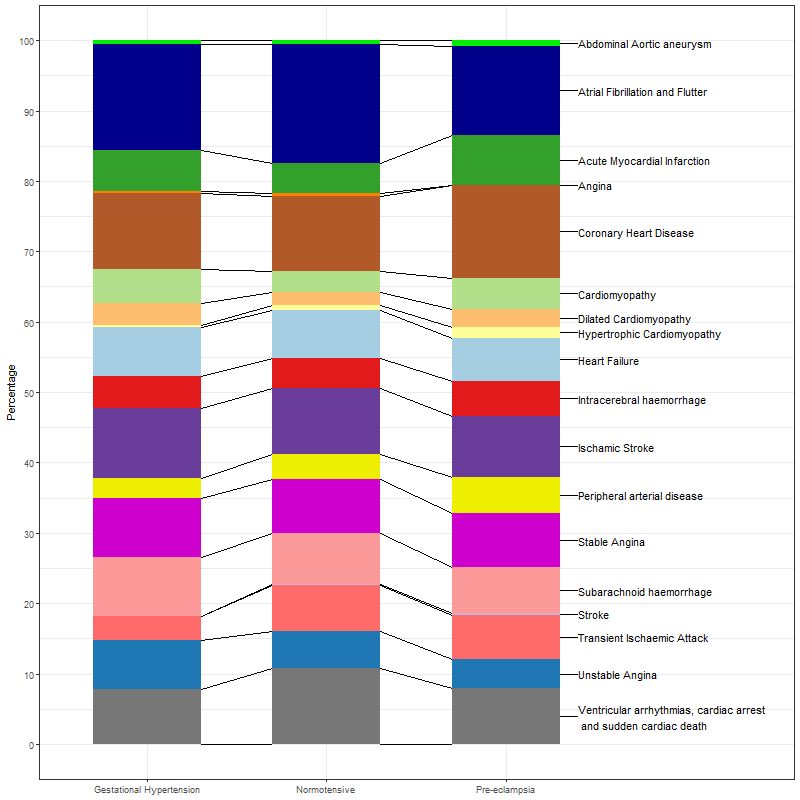


eFigure 5: Cardiovascular disease risk more than 6 months after first delivery of a pregnancy complicated by gestational hypertension compared to women with normotensive pregnancies over time, England, 1997-2015

*Adjusted for age and calendar year*

eFigure 6: Cardiovascular disease risk more than 6 months after first delivery of a pregnancy complicated by pre-eclampsia compared to women with normotensive pregnancies over time, England, 1997-2015

*Adjusted for age and calendar year*


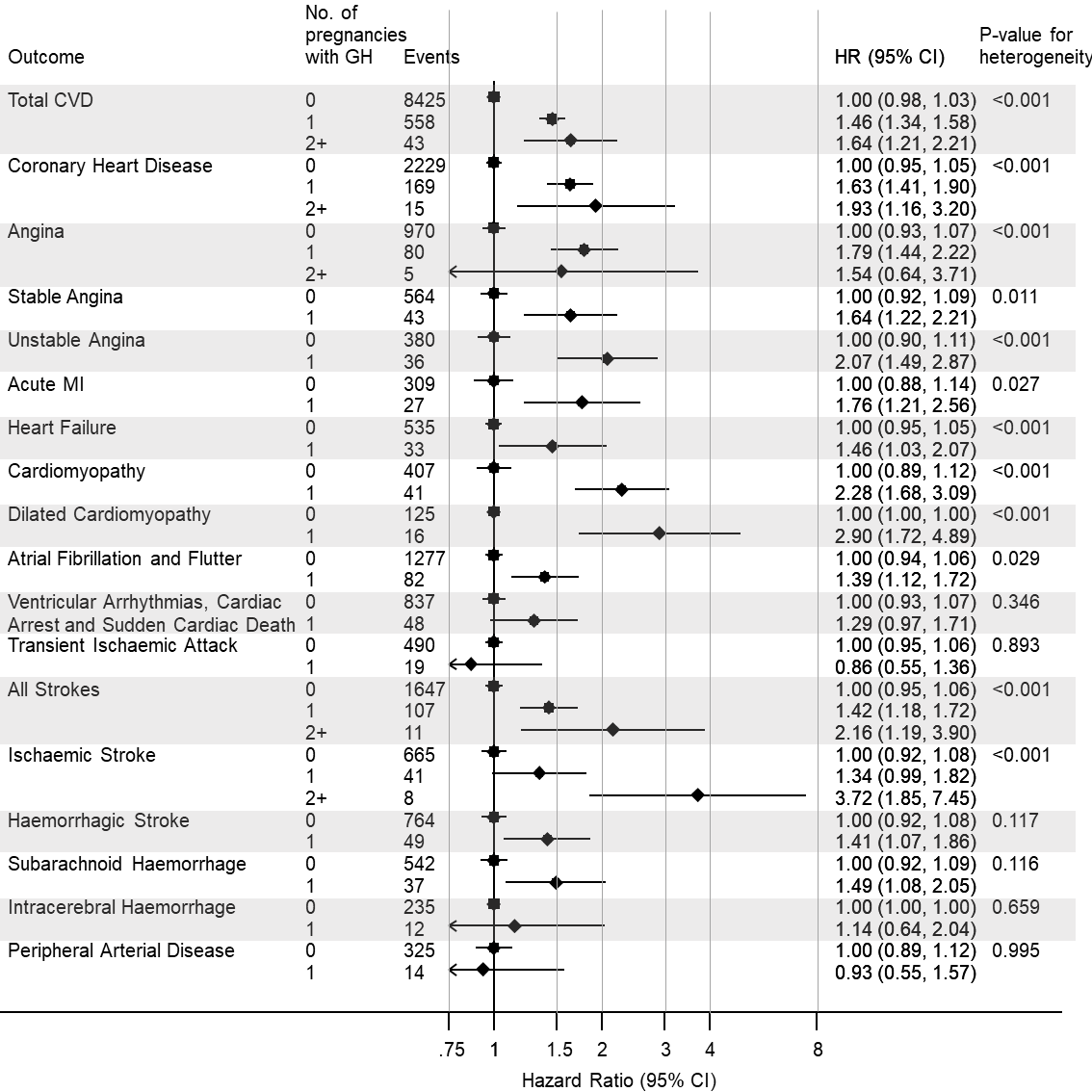


eFigure 7: Hazard Ratios for risk of pre-specified cardiovascular subtypes more than 6 months after first delivery by the number of pregnancies complicated by gestational hypertension compared to women with normotensive pregnancies, England, 1997-2015

*Adjusted for maternal age at delivery, socioeconomic status, ethnicity and diabetes*


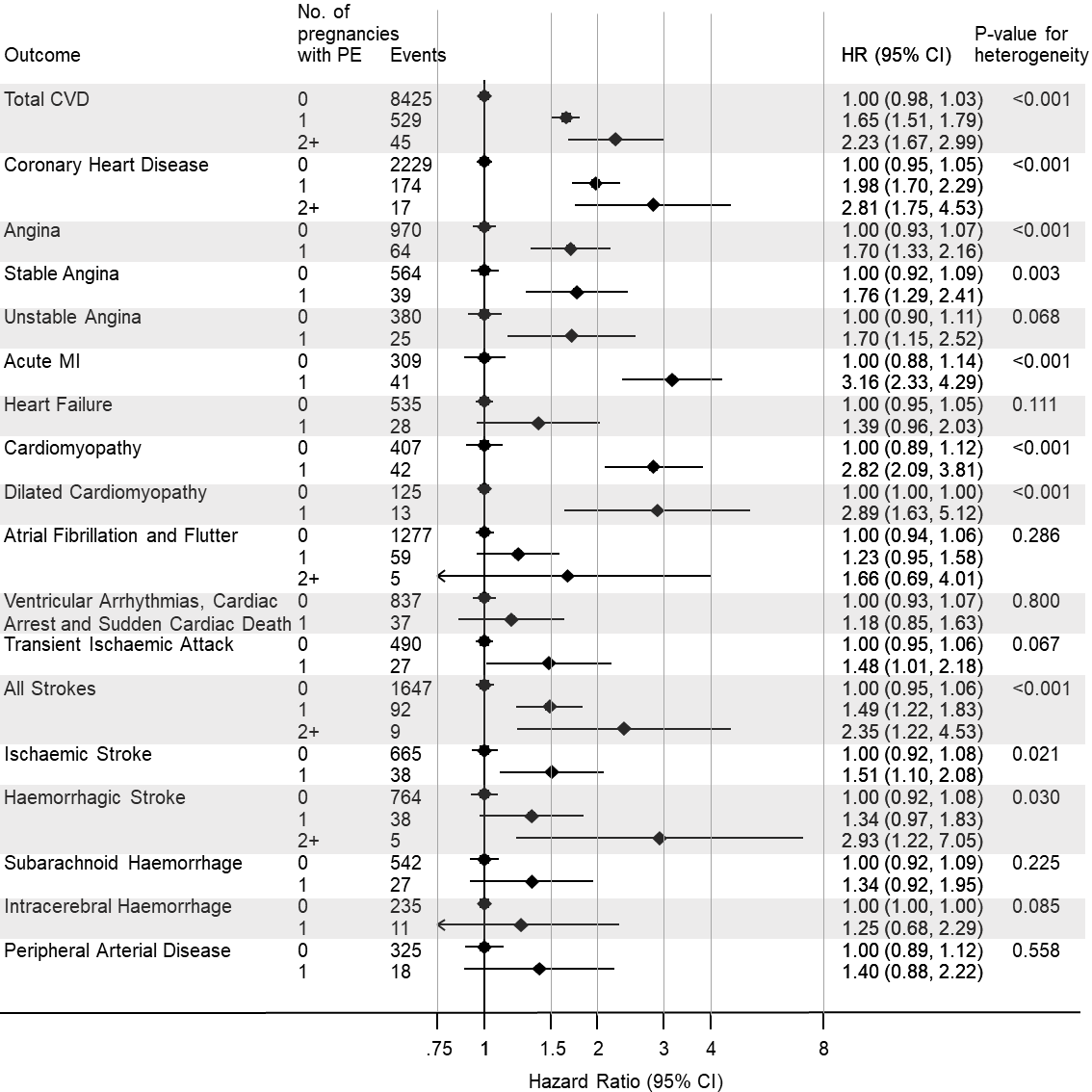


eFigure 8: Hazard Ratios for risk of pre-specified cardiovascular subtypes more than 6 months after first delivery by the number of pregnancies complicated by pre-eclampsia compared to women with normotensive pregnancies, England, 1997-2015

*Adjusted for maternal age at delivery, socioeconomic status, ethnicity and diabetes*


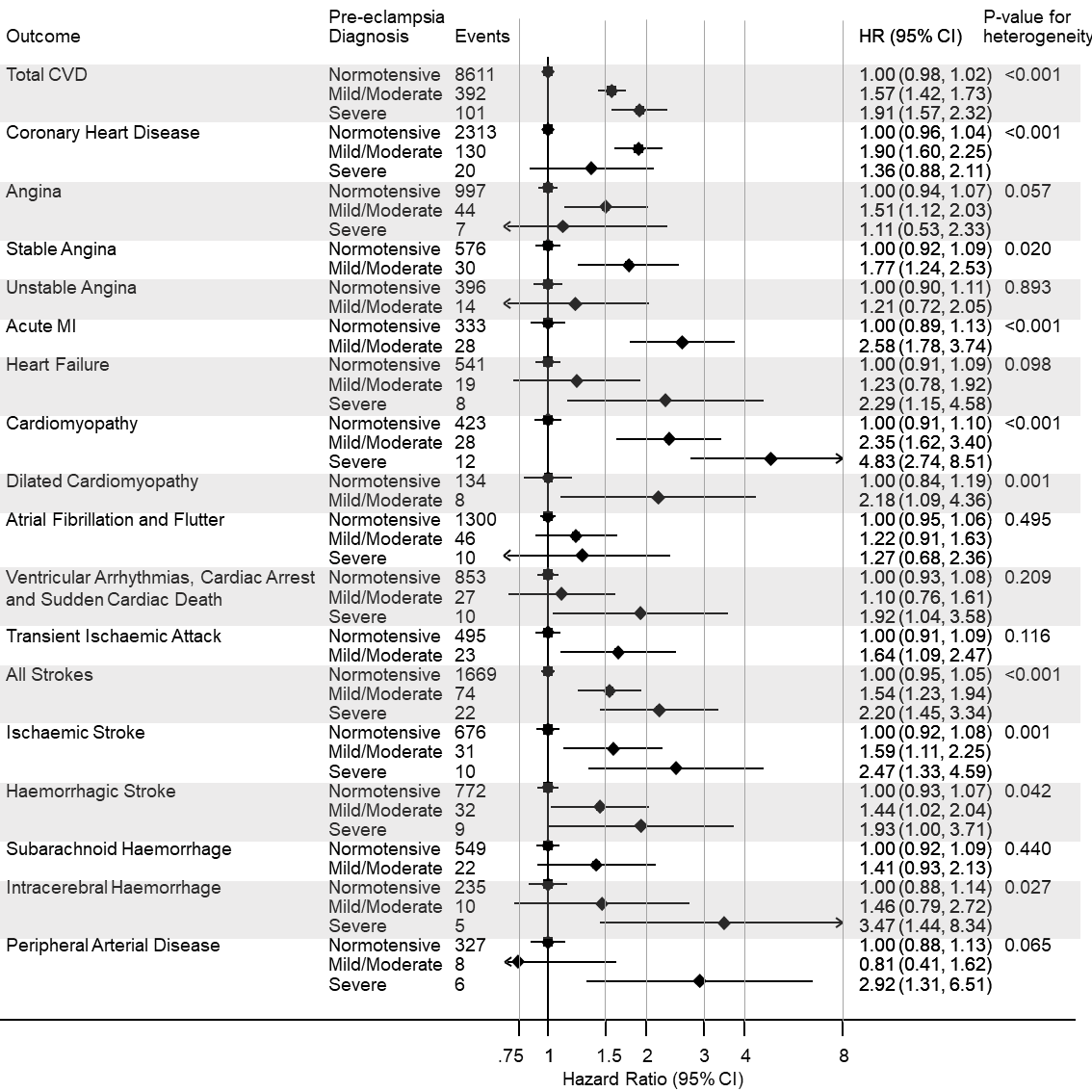


eFigure 9: Hazard Ratios for risk of pre-specified cardiovascular subtypes more than 6 months after first delivery of a pregnancy complicated by mild pre-eclampsia and severe pre-eclampsia compared to women with normotensive pregnancies, England, 1997-2015

*Adjusted for maternal age at delivery, socioeconomic status, ethnicity and diabetes*
